# A systematic review of the role of quantitative CT in the prognostication and disease monitoring of interstitial lung disease

**DOI:** 10.1183/16000617.0194-2024

**Published:** 2025-04-30

**Authors:** Giles Dixon, Hannah Thould, Matthew Wells, Krasimira Tsaneva-Atanasova, Chris J. Scotton, Michael A. Gibbons, Shaney L. Barratt, Jonathan C.L. Rodrigues

**Affiliations:** 1 Department of Clinical and Biomedical Sciences, University of Exeter, Exeter, UK; 2 Bristol Interstitial Lung Disease Service, North Bristol NHS Trust, Bristol, UK; 3 South West Peninsula ILD Network, Royal Devon University Healthcare NHS Foundation Trust, Exeter, UK; 4 Royal United Hospitals Bath NHS Foundation Trust, Bath, UK; 5 Academic Respiratory Unit, University of Bristol, Bristol, UK; 6 Department of Mathematics and Statistics, Faculty of Environment, Science and Economy, University of Exeter, Exeter, UK; 7 EPSRC Hub for Quantitative Modelling in Healthcare, University of Exeter, Exeter, UK; 8 Living Systems Institute, University of Exeter, Exeter, UK; 9 NIHR Exeter Biomedical Research Centre, Exeter, UK; 10 Department of Health, University of Bath, Bath, UK

## Abstract

**Background:**

The unpredictable trajectory and heterogeneity of interstitial lung disease (ILDs) make prognostication challenging. Current prognostic indices and outcome measures have several limitations. Quantitative computed tomography (qCT) provides automated numerical assessment of CT imaging and has shown promise when applied to the prognostication and disease monitoring of ILD. This systematic review aims to highlight the current evidence underpinning the prognostic value of qCT in predicting outcomes in ILD.

**Methods:**

A comprehensive search of four databases (Medline, EMCare, Embase and CINAHL (Cumulative Index to Nursing and Allied Health Literature)) was conducted for studies published up to and including 22 November 2024. A modified CHARMS (CHecklist for critical Appraisal and data extraction for systematic Reviews of prediction Modelling Studies) checklist was used for data extraction. The risk of bias was assessed using a Quality in Prognostic Studies template.

**Results:**

The search identified 1134 unique studies, of which 185 studies met inclusion and exclusion criteria. Commonly studied ILD subtypes included idiopathic pulmonary fibrosis (41%, n=75), mixed subtypes (26%, n=48) and systemic sclerosis ILD (16%, n=30). Numerous studies showed significant prognostic signals, even when adjusted for common covariates and/or significant correlation between serial qCT biomarkers and conventional outcome measures. Heterogenous and nonstandardised reporting methods meant that direct comparison or meta-analysis of studies was not possible. Studies were limited by the use of retrospective methodology without prospective validation and significant study attrition.

**Discussion:**

qCT has shown efficacy in the prognostication and disease monitoring of a range of ILDs. Hurdles exist to widespread adoption including governance concerns, appropriate algorithm anchoring and standardisation of image acquisition. International collaboration is underway to address these hurdles, paving the way for regulatory approval and ultimately patient benefit.

## Shareable abstract

**Quantitative CT (qCT) uses computer algorithms to extract prognostic and disease monitoring metrics from CT images. qCT has shown significant efficacy in the prognostication and disease monitoring of a broad range of interstitial lung diseases.** https://bit.ly/3EQHoQa

## Introduction

The interstitial lung diseases (ILDs) are a heterogenous group of lung conditions with varying degrees of inflammation and scarring (fibrosis). ILD progression is unpredictable, making prognostication challenging and creating barriers to timely use of disease modifying treatments, referral for lung transplantation and the introduction of supportive and palliative treatments. Lack of universally accepted criteria to define disease progression in ILD also contributes to difficulties in prognostication [1, 2].

Several models combining physiological and/or demographic variables, including the Gender Age Physiology (GAP) index and the Composite Physiological Index, have shown good prognostic performance across a range of fibrotic ILDs but are limited by physiological measurement variability, patient factors such as cough or fatigue, and variation in patient technique [3, 4]. Change in the physiological marker of forced vital capacity (FVC) remains the Food and Drug Administration (FDA) approved outcome measure for treatment trials in ILD, despite the aforementioned limitations [5]. The need for new primary end-points for clinical trials and clinical decision making has led to interest in alternate end-points such as 6-min walk distance (6MWD), patient reported outcome measures (PROMs) and quantitative radiological assessment [6].

High-resolution computed tomography (HRCT) is routinely used in patients presenting with ILD and captures the disease extent and morphology. However, HRCT can have limitations such as respiratory motion artefacts and inconsistent depth of inspiration. Furthermore, visual assessment of computed tomography (CT) patterns is limited by time constraints, the availability of subspecialty expertise and susceptibility to inter-observer bias [7–10]. Quantitative CT (qCT) employs a range of computer-based algorithms to objectively assess radiological features (table 1). qCT techniques can vary from simple statistical analysis of pixel histograms to utilising unsupervised convolutional neural networks to identify radiomic biomarkers that cannot be quantified using standard visual assessment (table 2) [11]. Despite much promise over the last two decades, qCT techniques have failed to gain widespread clinical or trial uptake.

**TABLE 1:** Glossary of terms [11, 129–131]

**Artificial intelligence**	The design and study of machines that can perform tasks that would previously have required human (or other biological) understanding to accomplish
**Convolutional neural network**	Deep learning algorithm used for image recognition tasks
**Feature**	Individual measurable property or characteristic
**Quantitative CT**	The use of computer algorithms to extract numerical outputs from a computed tomography (CT) image or images
**Radiomics**	A collection of techniques to extract quantitative features from imaging
**Segmentation**	Process of detecting the margins of a structure within an image
**Supervised learning**	An algorithm trained from labelled input data with the resulting model being tested to apply labels to new unlabelled data
**Support vector machine**	Supervised machine learning algorithm which classifies data by finding an optimal line or hyperplane that maximises the distance between classes in an N-dimensional space
**Unsupervised learning**	An algorithm trained using large volumes of unlabelled data in which novel patterns or clusters are automatically recognised without human supervision

**TABLE 2:** Commonly reported quantitative computed tomography (CT) techniques and algorithms

Algorithm	Description
**AirQuant**	Airway segmentation algorithm which calculates airway lumen diameter, length and branching points using a deep learning model to derive quantitative features of airway morphology [132]
**CALIPER (Computer-Aided Lung Informatics for Pathology Evaluation and Rating)**	Machine-learning algorithm that uses voxel histogram signatures to algorithmically identify and quantify five radiological features, including normal lung, emphysema, ground-glass density, reticular abnormalities and honeycombing CALIPER also extracts vessel-like structures, also known as pulmonary vessel volume [16]
**DTA (data-driven texture analysis)**	Deep-learning algorithm developed using unsupervised feature learning to build convolutional neural networks to quantify the percentage of lung pixels classified as fibrotic [43]
**e-Lung (Brainomix)**	Machine-learning based algorithm which quantifies a weighted reticulovascular score incorporating reticulation and peripheral vascular structure [133]
**Fibresolve**	Convolutional neural network designed to assess patients with suspected idiopathic pulmonary fibrosis and predict diagnosis [134]
**FRI (functional respiratory imaging)**	Algorithm which uses computational flow dynamics to perform flow simulations within the airways and pulmonary vessels in inspiration and expiration [135]
**GHNC (Gaussian histogram normalised correlation)**	Technique that classifies pixels using CT attenuation values into normal (N), ground-glass opacities (G), consolidation (C), emphysema (E) and fibrosis (F) patterns [136]
**Lung8, Fibr8, Vascul8 (Qureight)**	Airway, fibrosis and vascular markers based upon three-dimensional convolutional neural networks initially trained using a supervised approach [53, 137, 138]
**QLF (quantitative lung fibrosis)**	Machine-learning algorithm which quantifies the extent of reticulation and architectural distortion using a support vector machine classifier [17]
**SOFIA (Systematic Objective Fibrotic Imaging Analysis Algorithm)**	Convolutional neural network which classifies high-resolution CT into usual interstitial pneumonia probability scores [51]

We aimed to perform a systematic review of the literature to examine the role of qCT in the prognostication and disease monitoring of ILD. Our goal was to define the current scope of understanding and describe the future research priorities required to enable integration into routine clinical care.

## Methods

**FIGURE 1:**
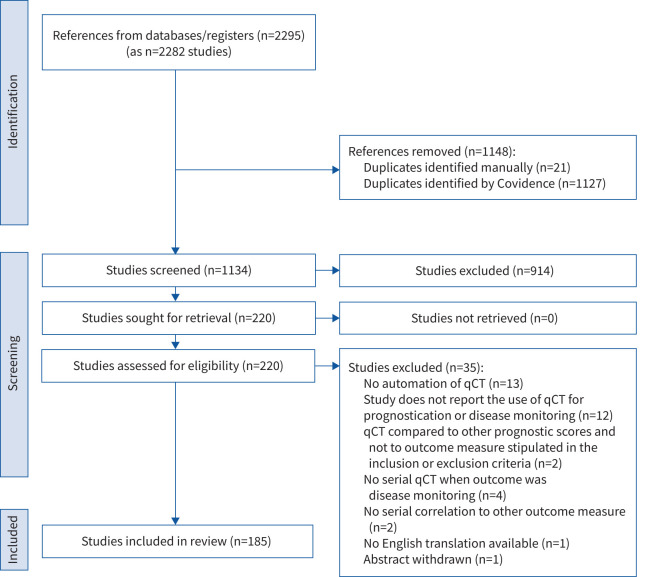
PRISMA (Preferred Reporting Items for Systematic Reviews and Meta-Analyses) diagram for a systematic review of the role of quantitative computed tomography (qCT) in the prognostication and disease monitoring of interstitial lung disease.

This systematic review was conducted and reported in accordance with the Preferred Reporting Items for Systematic reviews and Meta-Analyses (PRISMA) reporting guidelines [12] (figure 1 and supplementary information 1). The study protocol was registered in the PROSPERO database before the initiation of the study (PROSPERO ID CRD42022384186).

### Search strategy

A comprehensive search strategy was developed in collaboration with North Bristol NHS Trust Library and Knowledge services (supplementary information 2). The search, limited to articles in English was undertaken on 22 November 2024 in Medline (1946 to search date), EMCare (1995 to search date), Embase (1974 to search date) and CINAHL (Cumulative Index to Nursing and Allied Health Literature) (1984 to search date). A grey literature search was performed of the reference lists of the identified studies, systematic reviews, reviews, guidelines or highlighted by experts in the field.

### Study selection

All citations retrieved in the search were uploaded into Covidence (Covidence systematic review software, Melbourne, Australia) and duplicate records were removed. We included prospective and retrospective studies for adults ≥18 years old where qCT software had been investigated for the prognostication or monitoring of ILD. Disease outcomes included (but were not limited to) the ability of baseline qCT to predict disease progression, all-cause mortality and progression-free survival. Articles which used existing prognostic scores as outcome measures (*e.g.* predicting GAP score) were excluded. Disease-monitoring outcomes included, but were not limited, correlations of serial qCT with spirometry, gas transfer and 6MWD. Review articles, studies without longitudinal patient data, case reports, teaching documents, editorials, guidelines, study protocols and animal studies were excluded.

All titles and abstracts were screened against inclusion and exclusion criteria by two of three independent reviewers (G. Dixon, H. Thould and M. Wells). Where inclusion was not clear from the title and abstract, full texts were reviewed. Any discrepancies were resolved through additional discussion and consensus agreement. Full-text articles for potentially eligible studies were retrieved and independently screened for eligibility by two of three authors (G. Dixon, H. Thould and M. Wells).

### Data extraction, quality assessment and risk of bias

Data extraction and risk of bias was completed by a single reviewer (G. Dixon). A CHARMS (CHecklist for critical Appraisal and data extraction for systematic Reviews of prediction Modelling Studies) checklist modified for use in systematic reviews of prognostic factors was adapted for use in this study (CHARMS-PF) [13]. A descriptive synopsis with summary data was produced and results were summarised using narrative synthesis. A synthesis without meta-analysis approach was adopted as the recording of outcomes did not exhibit sufficient uniformity for meta-analysis [14]. Novel or pertinent findings from conference abstracts not reproduced in peer-reviewed literature were included in the narrative. Studies were grouped by aim (prognostication or disease monitoring) and then sub-type of ILD. The effect measure reported by each study was extracted. Risk of bias was assessed using the Quality in Prognostic Studies (QUIPS) checklist [15].

## Results

### Search results

The electronic literature search identified 2295 potential studies. Following removal of duplicates (1148 studies), 1134 abstracts were screened against inclusion and exclusion criteria. Following full-text review (220 studies), 185 studies were eligible for full data extraction (figure 1). Reasons for study exclusion (n=35) are detailed in figure 1.

### Study characteristics

**FIGURE 2:**
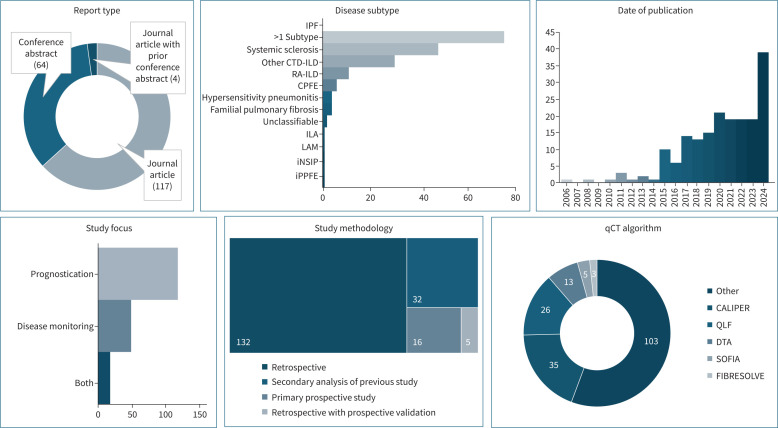
Visual summary of studies included in the systematic review. CALIPER: Computer-Aided Lung Informatics for Pathology Evaluation and Rating; CPFE: combined pulmonary fibrosis and emphysema; CTD-ILD: connective tissue disease–interstitial lung disease; DTA: data-driven textural analysis; ILA: interstitial lung abnormality; iNSIP: idiopathic nonspecific interstitial pneumonia; IPF: idiopathic pulmonary fibrosis; iPPFE: idiopathic pleuroparenchymal fibroelastosis; LAM: lymphangioleiomyomatosis; qCT: quantitative computed tomography; QLF: quantitative lung fibrosis; RA-ILD: rheumatoid arthritis related interstitial lung disease; SOFIA: Systematic Objective Fibrotic Imaging Analysis Algorithm.

Summary data for 185 included studies can be found in figure 2. The included studies (including conference abstracts) were published between 2006 and 2024. 65% (121/185) studies were available as journal articles (with or without associated conference abstracts) and 35% (64/185) were available as conference abstracts only. Data extraction was not possible from the 64 conference abstracts due to inconsistent reporting.

### Participant numbers

The number of participants included in final analyses ranged from 17 to 11 627, with a median and interquartile range of 118 and 181 participants, respectively. A total of 78 311 patients were screened for study inclusion and 65 075 patients were included in the analysis. Reasons for exclusion were not provided in 4108 cases. Where reported, 4540 patients were excluded due to missing data, 3151 due to incorrect CT scan acquisition protocol or parameters and 1437 due to co-existing lung disease.

### Study methodology

Most studies used retrospective methodology with propriety (65%, 121/185) or open-access databases (6%, 11/185) or secondary analysis of prospective studies (16%, 30/185) studies. Only 10% (19/185) of studies presented a study with primary prospective methodology.

### Quality assessment

Quality assessment for the risk of bias was conducted with the use of the QUIPS checklist (table S1a and S1b) [15]. 7% (8/121) of studies reported as journal articles had at least moderate risk of bias concerning study participation. This was predominantly due to small participant cohorts or inadequate description of inclusion and exclusion criteria. Study attrition was the main source of potential bias; 26% (32/121) studies had at least moderate risk of bias. Sources of attrition included inadequate matching of pulmonary function testing (PFT) with qCT, inadequate quality of imaging and co-existent disease. A limited number of studies had a moderate or higher risk of bias as regards prognostic factor measurement, outcome measurement, study confounding and statistical analysis and reporting. For example, studies may have inferred causation or prediction when demonstrating an association or alternatively presenting unadjusted hazard ratios when assessing an association. Limited assessment was made of articles available only in conference abstract form. 23% (15/64) of abstracts demonstrated a moderate or higher risk of bias in at least one domain. Overall, except for study attrition, the quality of the analysed literature was high with a low risk of bias.

### qCT software

**FIGURE 3:**
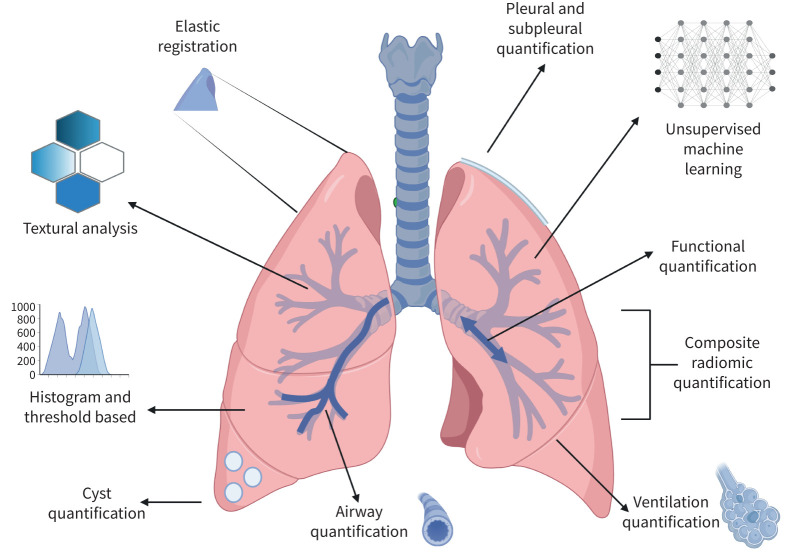
Methods of quantitative CT for the prognostication and disease monitoring of interstitial lung disease. Examples of relevant methods include: pleural and subpleural quantification [101], unsupervised machine learning [52, 56], functional quantification [19, 76], composite radiomic quantification [74, 115], ventilation quantification [70], airway quantification [46, 114], cyst quantification [87], histogram and threshold-based [32, 88, 139–142], textural analysis [16, 36, 37, 41, 143], and elastic registration [75, 144].

There were a range of quantitative techniques applied (table 2). Computer-Aided Lung Informatics for Pathology Evaluation and Rating (CALIPER) (19%, 35/185), quantitative lung fibrosis (QLF) (14%, 26/185) and data-driven textural analysis (DTA) 7%, 13/185) were the most commonly used quantitative algorithms [16–18]. qCT algorithms ranged from histogram or threshold-based analysis of lung density to manually engineered features. Deep learning was employed in several studies to extract radiomic features from datasets by unsupervised machine learning (ML). The features engineered included the quantification of airways, pulmonary vasculature, subpleural fibrosis and cysts; these are presented pictorially in figure 3.

### Idiopathic pulmonary fibrosis (IPF)

36% (44/121) studies available as journal articles exclusively included patients with IPF (table S2), including 37 retrospective and seven prospective studies. Several studies utilised clinical trial datasets from previously reported randomised control trials (RCTs); however, only a single study by Lancaster *et al.* [19] included qCT as a primary outcome measure [20–27]. Two studies utilised open-source imaging databases from the Open-Source Imaging Consortium (www.osicild.org) [28, 29].

Analysis of whole lung histogram was the earliest qCT technique described; this technique involved semi-automated lung field segmentation followed by analysis of the kurtosis, skewness and mean attenuation derived from attenuation histograms [30, 31]. Analysis of kurtosis showed promise for predicting mortality on univariate analysis (median follow up 1.5 years) however on multivariate analysis, visually derived fibrosis score significantly outperformed kurtosis (OR 1.104 *versus* 0.579, respectively). Several other studies reported the use of attenuation histogram statistics to predict mortality but are limited by their ability to identify individual morphological features and can be significantly impacted by the presence of emphysema [32, 33].

The development of novel software approaches and the enhanced processing power of hardware has led to computer vision techniques such as CALIPER which uses expert radiologist labelled volumes of pixels to train an algorithm to identify specific lung textures [16]. Maldonado *et al.* [16] reported the ability of serial change in CALIPER textures to predict survival over a median follow up of 2.4 years. This ML morphological analysis has subsequently been used in several studies of prognostication and disease monitoring [25, 34–39]. Quantitative analysis of pulmonary vessel volume (PVV) was reported by Jacob *et al.* [34] as being predictive of mortality on multivariate analysis (hazard ratio (HR) 1.53, 95% CI 1.41–1.66, p<0.0001) and showing improved stratification of patient prognoses compared to the GAP staging system. Changes in PVV also demonstrated a good correlation with changes in FVC (R^2^ =0.57) over a mean±sd interval of 1.1±0.4 years [36]. Exposure to ionising radiation is often discussed as a barrier to the routine use of qCT in disease monitoring, yet reassuringly CALIPER variables have demonstrated good concordance between CT scans performed at standard, low and ultra-low dose [37].

QLF utilises a support vector machine to derive the QLF score, which includes fibrotic reticulation patterns only, and quantitative ILD score (QILD), which includes honeycombing, ground glass and reticulation [40]. QLF scores in the worst-affected lobes are associated with a higher risk of progression, even when adjusted for baseline GAP score (HR±se 5.92±3.11, p=0.01) [41]. QLF has also demonstrated utility as a clinical trial outcome measure, demonstrating a correlation between changes in QLF and QILD with changes in FVC, diffusing capacity of the lung for carbon monoxide (*D*_LCO_) and PROMs (University of California San Diego Shortness of Breath Questionnaire) [19, 22]. Efforts to define a minimum clinically important difference (MCID) of QLF have been reported in abstract form, suggesting that anchored to FVC at 48 weeks, a 4.3% change in QLF may be a potential clinical trial end-point [42].

DTA uses unsupervised ML to identify clusters of low-level pixel patterns that can be searched for in regions of interest, such as expert-labelled usual interstitial pneumonia (UIP) patterns [18, 43]. DTA can predict referral to transplant and progression-free survival when adjusted for age, gender, body mass index (BMI), smoking and antifibrotic use. It also correlates with changes in FVC, *D*_LCO_, 6MWD and St George's Respiratory Questionnaire score [26, 43, 44].

Additional qCT methods have been employed, including functional respiratory imaging (FRI), which utilises computational fluid dynamics to model changes in the airway tree [20]. Airway radius has been proposed as a method to detect early progression not typically identified by FVC [20, 45]. The presence of airway changes, specifically traction airway dilatation, has shown promise on visual assessment and efforts to apply qCT to extract airway features have been reported [46–48]. AirQuant is a qCT algorithm which calculates airway lumen diameter and length and uses this to quantify intersegmental tapering, segmental tortuosity and total segment count [49]. Cheung *et al.* [46] demonstrated on multivariate cox analyses adjusted for FVC that segmental tortuosity was associated with mortality (HR 1.74, 95% CI 1.22–2.47, p=0.002, median follow-up 2.68 years). Alternative airway quantification techniques have demonstrated that quantification of airway walls of segmental and subsegmental airways are associated with mortality (HR 1.58, 95% CI 1.17–2.14, p<0.01) [50].

qCT developers are increasingly adopting unsupervised deep-learning techniques to extract radiomic biomarkers which are then mapped against an outcome [51]. In an Australian IPF Registry, the Systematic Objective Fibrotic Imaging Analysis Algorithm (SOFIA) UIP probability category was independently associated with survival (HR 1.29, 95% CI 1.17–1.41, p<0.0001) [52]. Furthermore, the SOFIA score demonstrated an association with progression at 12 months even when adjusted for radiologist-defined total ILD extent (OR 1.58, 95% CI 1.28–1.85, p<0.0001).

Most recently, Thillai *et al.* [53] reported the use of fibrosis, airway and vascular markers in the prospective PROFILE cohort and validated in a separate retrospective UK cohort. The study demonstrated association between the three markers and 2-year progression-free survival after adjustment for baseline GAP score. Furthermore, serial lung and fibrosis volume changes were shown to be associated with differential survival.

Alternate quantitative techniques have been proposed, including mapping lung shrinkage whereby elastic registration techniques are applied to serial HRCT and the quantification of mediastinal adipose tissue to predict mortality and disease progression [54, 55]. Novel methods of training networks to predict outcome (disease progression or transplant-free survival) have been developed. Pan *et al.* [56] assessed candidate radiomic biomarkers against their ability to recognise the temporal sequence of examinations, exploiting the irreversible nature of fibrosis. Shi *et al.* [24] reported using a wrapper method known as quantum particle swarm optimisation to search computational high-dimensional spaces for a small number of high-performing features to predict mortality.

Combining radiomic biomarkers with clinical data can build effective disease progression prediction models. Bak *et al.* [57] used cluster-based analysis combining radiomics with clinical variables such as age, sex and PFT, and then demonstrated different prognoses between separate clusters. Budzikowski *et al.* [27] offered preliminary analysis to suggest the combination of genetic mutation signatures with radiomic data may offer multiple different Kaplan–Meier survival curves.

The majority of studies selected mortality or progression free survival as an outcome measure. Wang *et al.* [58] presented the use of AView software to quantify lung textures and demonstrated that honeycombing and whole lung volume were independently associated with acute exacerbations of IPF in a prospective cohort of 102 patients.

### Connective tissue disease (CTD) ILD

27% (33/121) of studies available as journal articles were conducted in a population of patients with CTD-ILD (table S3). Systemic sclerosis (SSc) ILD was the most investigated condition (58%, 19/33 studies) followed by anti-melanoma differentiation-associated protein 5 (MDA5) dermatomyositis (12%, 4/33 studies), rheumatoid arthritis (RA) ILD (2%, 4/33 studies) and idiopathic inflammatory myopathies (9%, 3/33 studies). The remaining 4/33 studies investigated several CTD-ILD subtypes simultaneously. Most studies used retrospective methodology (27 studies) with only six studies having prospective validation. CALIPER (8/33 studies) and QLF (6/33 studies) were commonly used with the remaining studies using varied qCT techniques. 18 studies focussed on prognostication alone, 12 on disease monitoring and three studies on both prognostication and disease monitoring.

Five studies assessed qCT in CTD-ILD RCTs including Scleroderma Lung Study (SLS) I (three studies) and SLS II (one study) and a trial of dasatinib in SSc-ILD [59–63]. Four studies applied QLF retrospectively to prospective interventional clinical trials. Goldin *et al.* [59] reported a correlation between changes in QILD score and FVC in the SLS II trial of cyclophosphamide *versus* mycophenolate mofetil (r=0.37, p<0.001). The three studies assessing the SLS I study reported different uses of qCT. Kim *et al.* [61] reported a correlation between changes in QLF score and changes in FVC (r=−0.33, p=0.003). Khanna *et al.* [62] demonstrated in the placebo arm of SLS I that high baseline fibrosis was associated with greatest FVC decline. Volkmann *et al.* [63] generated a composite outcome measure including FVC% predicted, QLF-ZM (QLF-score in zone of maximal involvement), transitional dyspnoea index and health assessment questionnaire–disability index. The composite outcome measure demonstrated a significant treatment effect favouring cyclophosphamide (estimate±se 0.7±0.2, p=0.005) [63]. One study prospectively included QLF as a secondary outcome measure and demonstrated a correlation between changes in QILD in the most severely affected lobe *versus D*_LCO_ (r=0.69, p<0.0001) [60].

QLF was also used in RA-ILD whereby Lee *et al.* [64] demonstrated a correlation between an increase in QILD score and radiologist visual assessment of progression in a retrospective and prospective cohort of patients. Baseline CALIPER measures including ground glass opacities, PVV and total lung volume were associated with reductions in *D*_LCO_, mortality and composite respiratory end-points, respectively, in SSc-ILD, undifferentiated CTD-ILD and RA-ILD [65–68]. Changes in total interstitial lung abnormality (ILA) derived by CALIPER was also shown to correlate with changes in *D*_LCO_ (r=–0.293, p=0.05) [69].

qCT was also applied to inpatient cohorts of patients with anti-MDA5 dermatomyositis to quantify consolidation, effective lung ventilation area ratio (ELVAR) and extent of lung involvement (radiomic and lung severity scores) [70–73]. Xu *et al.* [71] reported two studies with C-indices ranging from 0.64 to 1.0 for the prediction of 6-month mortality from qCT employed in hospitalised patients [72]. Wang *et al.* [70] reported ELVAR as a method of quantifying pulmonary ventilation function and demonstrated an association with 12-month mortality on multivariate analysis (HR 0.098, 0.017–0.0564, p=0.009). Conversely, a lung severity score was developed by Yamaguchi *et al.* [73] which had an area under the curve of 0.844 (p<0.01) for the prediction of early mortality (median 35 days).

In a manner similar to studies on IPF, Qin *et al.* [74] looked to combine clinical and radiomic features to predict survival in SSc-ILD. A model including three radiomic features and three visually extracted CT features (traction bronchiectasis, visual ILD % change and visual ILD volume change) demonstrated greater ability to predict overall survival *versus* a clinical (visual) model alone (C-indices 0.74–0.80 *versus* 0.59–0.75) in a training, internal validation and external validation cohort.

Alternative reported qCT methods include elastic registration by Chassagnon *et al.* [75], who demonstrated a correlation between log_jac values and *D*_LCO_ (r=−0.42, p<0.001). Clukers *et al.* [76] also employed FRI to quantify airway dynamics, demonstrating that specific imaged-based airway radius (siRADaw) showed a significant reduction in a cohort of patients retrospectively classified into moderate to severe lung disease *versus* no significant reduction in limited lung disease.

A primary and validation cohort of patients with RA-ILD was recently reported by Humphries *et al.* [77], who demonstrated a correlation between longitudinal FVC and *D*_LCO_ with a DTA fibrosis score over a median follow-up between scans of 401 days (r=−0.46 and −0.43, p<0.001 respectively). DTA score was also independently associated with mortality risk when adjusted for age, sex, BMI, disease-modifying anti-rheumatic drug use and smoking history.

### Other ILD subtypes

qCT has also been utilised in 36% (44/121 studies) of other sub-types of ILD, in addition to combined cohorts of patients, demonstrating the efficacy of radiomic features across a range of ILDs (table S4).

In combined pulmonary fibrosis and emphysema (CPFE), the combination of emphysema and fibrosis limits the use of FVC to monitor progression [78]. Zhao *et al.* [79] demonstrated the use of an ML algorithm to delineate two distinct populations of patients with CPFE. They demonstrated that FVC progression was a poor mortality surrogate in an ML-derived “matched-CPFE” cohort with co-existent fibrosis and emphysema at baseline, whilst demonstrating that *D*_LCO_ reduction in this population remained a valid measure of prognostication. Other studies demonstrated that CALIPER-derived fibrosis extent was predictive of death or disease progression (HR 6.85, 95% CI 2.85–15.89, p<0.001) and histogram-derived percentage abnormal area (a combination of low and high attenuation areas) was associated with survival (OR 1.2, 1.02–1.54, p=0.029) [80, 81]. Despite significant emphysema, Ando *et al.* [82] were able to demonstrate a correlation between parenchymal density and FVC decline in a small cohort of patients with CPFE (r=0.714, p=0.047).

Beyond the work in IPF, Jacob *et al.* [83] has also demonstrated the predictive value of the CALIPER-derived features of reticulation and PVV in subacute and chronic hypersensitivity pneumonitis (HP) survival (HR 0.95, 95% CI 0.93–0.97, p<0.0001 and HR 1.74, 1.31–2.31, p<0.0001, respectively) [84]. Meanwhile, Aliboni *et al.* [85] demonstrated a correlation between fibrosis extent and FVC decline over a median of 1.4 years (R^2^=0.54, p<0.001) in a small retrospective cohort of HP patients. Three-dimensional (3D) CT-derived lung volumes have been used in fibrotic HP in combination with antigen exposure status to create a prognostic score able to classify patients into distinct mortality risk categories [86]. Despite this progress, no studies have prospectively examined the role of qCT in HP.

Other subtypes of ILD have also been examined, including two studies that investigated qCT in quantifying disease progression in lymphangioleiomyomatosis [87, 88]. Argula *et al.* [87] reported no correlation between qCT-derived lung cyst volume or number with change in FVC, forced expiratory volume in 1 s or serum vascular endothelial growth factor-D, but did suggest that the observed variation in respiratory cycle cyst volume demonstrated the beneficial effect of sirolimus on airflow obstruction, highlighting a role for qCT in mechanistic studies. A single study by Fukada *et al.* [89] quantified upper lobe volumes using 3D-CT in pleuroparenchymal fibroelastosis (PPFE) and demonstrated a significant association with mortality, despite adjusting for age and sex. In patients with idiopathic nonspecific interstitial pneumonia, Salhofer *et al.* [90] demonstrated the quantification of a pulmonary fat index is associated with mortality independent of BMI and other common comorbidities (HR 2.37, 95% CI 1.03–5.48, p=0.043).

In a UK retrospective cohort of patients with unclassifiable-ILD, Jacob *et al.* [91] showed PVV to be a strong predictor of outcome on univariate analysis; however, multivariate analysis was not possible due to collinearity. Visual-derived quantification of traction bronchiectasis and pulmonary artery diameter, however, independently predicted mortality (p=0.003 for both features). Despite the high prevalence of unclassifiable ILD, the study by Jacob *et al.* [91] was the only included study to investigate this cohort of patients.

Familial interstitial pneumonia has been a further area of study and identifying early disease in relatives at risk of pulmonary fibrosis may enable early disease intervention. Steele *et al.* [92] reported the use of DTA in a cohort of 493 asymptomatic first-degree relatives, with 296 undergoing serial evaluation. Baseline Quantitative Fibrosis score was associated with worsening breathlessness over a median 3.9 years of follow-up, as well as being independently associated with decreased survival (HR 1.06, p=0.01) [92]. The authors suggest that qCT could be used as a method of risk stratification when determining the monitoring of relatives at risk of developing ILD.

### ILAs

ILAs are any incidental CT pattern that exceeds 5% in any single lung zone and can be associated with the development of ILD [93]. Chae *et al.* [94] reported the use of textural analysis to identify ILAs in a Korean lung cancer screening population. A 1.8% threshold in quantitative fibrosis had a 100% sensitivity and 99% specificity for the identification of visually defined ILA. Those with progressive disease, defined by visual assessment, demonstrated a greater increase in quantitative assessment than those with stable disease (3.1% *versus* 0.1%, p<0.001). In a similar population, QLF-identified ILAs were associated with poorer 5-year survival in a lung cancer screening cohort (3.4% *versus* 1.5% mortality, p=0.010) [95]. In the prospective Multi-Ethnic Study of Atherosclerosis (MESA) study of 6814 adults, Choi *et al.* [96] showed that doubling of lung high-attenuation areas on serial CT was associated with a higher risk of ILD-specific mortality (HR 3.30–3.98, p<0.001) and lower FVC in specific zonal distributions (113–186 ml, p<0.001). These data suggest qCT may have a role in the identification and stratification of patients within screening programmes.

### Mixed ILD subtypes

25 studies reported the use of qCT in cohorts of mixed ILDs. A study by Oh *et al.* [97] reported the use of DTA in patients recruited to the Pulmonary Fibrosis Foundation Patient Registry. In a population of 979 patients with a range of ILDs, the extent of lung fibrosis defined by DTA was independently associated with transplant-free survival (HR 1.04, 95% CI 1.04–1.05, p<0.001). Identifying prognostic factors independent of diagnosis is a valuable tool, particularly given the lack of consensus between multidisciplinary teams for some subtypes of ILD [8]. Fibresolve, an algorithm built using a convolutional neural network, has been shown in separate studies to be associated with mortality even when adjusted for GAP score and other variables in cohorts with a broad range of ILD subtypes [98, 99]. In a comprehensive study using four different populations Humphries *et al.* [100] showed that multiple instance learning-UIP (MIL-UIP) score was associated with a greater annual decline in FVC even when adjusted for extent of lung fibrosis (−88 mL·year^−1^ *versus* −45 mL·year^−1^, p<0.01). Beyond the identification of UIP-like signals, Gudmundsson *et al.* [101] developed an algorithm to quantify visceral pleural surface affected by PPFE in patients with alternate diagnoses. Changes in the combined PPFE score were independently associated with mortality (HR 1.25, 95% CI 1.16–1.34, p<0.0001) and correlated with changes in FVC (R^2^=0.07, p<0.0001) [101].

Other novel applications showed the potential for combined radiomic and clinical models. Mei *et al.* [102] developed a dynamic model that can predict survival based on longitudinal clinical and radiomic data. The ability of the model to dynamically react to new longitudinal data demonstrated a clear clinical application, with the model's performance improving over time. An important negative study by Zou *et al.* [103] highlighted limitations in feature-derived qCT where CALIPER HRCT metrics showed little to no correlation with the slope of FVC decline in two treatment trials of IPF and SSc-ILD, respectively. Feature-derived metrics did, however, show promise in other populations including fibrosis scores, CT-derived lung volumes, CALIPER-derived ground glass, honeycombing and reticulation [104–106]

Pulmonary hypertension (PH) can be associated with ILD and is associated with reduced functional status and increased mortality [107]. Alkhanfar *et al.* [107] demonstrated that small PVV is significantly reduced in patients with PH-associated ILD and those patients with a lower volume had higher mortality. Mapping lung perfusion was also reported using a dual-energy CT, which differentiates iodine contrast from lung tissues, enabling the assessment of perfusion within morphologically abnormal areas [108]. Moon *et al.* [109] showed that the mean iodine value for the whole lung was associated with mortality in multivariate analysis adjusted for age and sex (HR 1.19, 95% CI 1.01–1.40, p=0.04).

### Studies available in abstract form only

35% (64/185) studies were available in abstract form only. Abstracts of note include Fang *et al.* [110], who reported the use of an IPF deep-learning algorithm that was independently associated with mortality even when adjusted for visual-based total fibrosis extent (HR 1.03, p<0.0001). Elsewhere, Kim *et al.* [111] showed a correlation between serial QLF score and King's Brief Interstitial Lung Disease questionnaire score, demonstrating an association between quality-of-life scoring and qCT. Fernandez Perez *et al.* [112] presented data from the PREDICT-HP multicentre observational cohort of patients with HP demonstrating that baseline DTA score is associated with a decrease in progression-free survival (HR 1.03 per % point in fibrosis score, 95% CI 1.01–1.05, p=0.005). Prospective cohorts such as PREDICT-HP may be best placed to demonstrate the role of qCT in routine clinical practice. Finally, Wang *et al.* [113] analysed National Lung Screening Trial data, showing that DeepLTA can identify features of ILD in a cancer screening population that are independently associated with mortality.

## Discussion

The present review highlights the evolution of qCT algorithms over the past two decades, from histogram-based analysis of whole lung density through supervised ML approaches generating textural analysis, to unsupervised deep-learning techniques that identify radiomic biomarkers which perform independently of visual CT analysis. The studies presented have demonstrated robustly that qCT biomarkers can accurately predict prognosis even when adjusted for other commonly used tools and covariates such as GAP index, visually identified radiological features and circulating blood biomarkers [44, 52, 53, 100, 114, 115]. Studies have also demonstrated a close correlation between PFTs, PROMs and visual changes with qCT biomarkers in clinical trials, prospective datasets and extensively in retrospective cohorts [21, 41, 101].

Despite the progress and breadth of evidence, the routine use of qCT has yet to be adopted into routine clinical use or as a regulatory-approved primary outcome measure for drug development. The reasons for this delay are complex and have been highlighted by several recent position papers [116, 117]. International efforts have been established to standardise CT acquisition, data governance and provide high-quality open access data for biomarker discovery. Consortia such as the Open-Source Imaging Consortium aim to advance the pipeline of imaging biomarkers into routine clinical adoption [117]. Efforts such as these aim to overcome the limitations of the current evidence and address the hurdles preventing widespread adoption.

A prominent limitation highlighted over the course of the review is the use of retrospective, proprietary databases for the training or validation of datasets. The CT scans collected in these studies are often performed where a suspicion of clinical disease progression is already present. Visually defined progression of radiological findings in combination with clinical symptoms is a widely accepted and used criteria for the diagnosis of progressive pulmonary fibrosis but is known to be insensitive to small changes in disease [10, 118, 119]. Algorithms trained on data sets which by default contain CT scans weighted towards progression may inherently incorporate the insensitivity to detect small but clinically valuable changes in disease morphology. Training or validation of algorithms on routinely collected imaging as part of prospective observational or interventional trials would overcome these biases and efficacy has already begun to be demonstrated in this setting [41, 112].

Determining an MCID for qCT algorithms requires adequate anchoring to known and validated current outcome measures [116]. Anchoring qCT outcome measures against how a patient “feels, functions and survives” would ensure outcomes become meaningful to patients, an approach already demonstrated in other areas of respiratory medicine [120]. Several studies have demonstrated correlation with PROMs, but few have utilised these in the training of their algorithms [25].

Clinical adoption will require demonstration to clinicians that qCT can impact upon clinical decision-making and empower clinicians to understand how the metrics were generated. Disease monitoring is one area whereby an immediate clinical impact could be felt. The ability to pre-empt imminent functional decline could enable the early introduction or modification of current therapies such as antifibrotics or immunomodulation. Accurate prognosis could also enable the appropriate integration of palliative and supportive care early in the patient pathway.

Additional obstacles exist before the widespread adoption of qCT, including concerns regarding radiation exposure and standardisation of HRCT acquisition. The minimal lifetime risks from ionising radiation exposure are offset by the reduced life expectancy associated with fibrotic ILD and the advanced age of many patients. Furthermore, studies have already demonstrated promise for the use of qCT with low-dose CT (LDCT) [36]. The commercial availability of qCT algorithms is also currently very limited and will be hindering clinical roll-out. Once algorithms become commercially available, the limited existing head-to-head comparisons available will make algorithm selection more difficult for the clinician, therefore potentially stifling adoption [121].

Furthermore, as routine LDCT scanning for lung cancer screening is introduced, validating biomarkers in a screening population of LDCT could lead to early disease recognition [122]. In this asymptomatic population, being able to predict the outcome of subclinical disease will require robust prospective validation. Standardisation of imaging acquisition and reconstruction protocols must be achieved to enable biomarker reproducibility. The use of protocolised acquisition has been demonstrated in other qCT technologies and should be adopted in the context of qCT for fibrotic ILD [123]. Spirometry gated qCT may help to account for variable lung volume at the time of CT acquisition and reduce respiratory motion artefact [124, 125]. Near-term reproducibility of qCT biomarkers has been demonstrated in limited qCT algorithms and highlights an important step in radiomic biomarker validation [126].

Beyond prognostication and disease monitoring qCT, the studies assessed in this review have highlighted other uses of radiomic biomarkers. qCT has a role in unpicking the mechanisms driving ILD and novel techniques combining qCT with gene expression and proteomic data have been demonstrated [115]. QCT may also have a role in predicting response to therapies or classification of disease patterns [127]. Ultimately, the integration of qCT into multi-omics disease models may reveal insights into ILD enabling mechanistic understanding and drug development.

### Limitations

The review has several limitations. The heterogenous reporting methods and the lack of standardised reporting tools meant direct comparison or meta-analysis of studies was not possible. Study attrition led to a moderate or higher risk of bias in at least 25% of studies reported as journal articles. Many studies were limited to retrospective datasets without real-world testing. Studies reporting segmentation and feature extraction dependent on lung density can be limited by CT dose, slice thickness and reconstruction kernel [128]. Multicentre standardisation is required to eliminate this important source of bias.

## Conclusion

The use of qCT in the prognostication and disease monitoring of fibrotic ILD has been extensively investigated and reported. Multiple high-quality studies have already demonstrated the efficacy of these new biomarkers; however, several hurdles remain for their adoption. Defining MCIDs anchored to outcome measures centred upon patients will help ensure tangible patient benefit. As the future of ILD looks towards early diagnosis and intervention, the ability of qCT to identify those at risk of disease progression will become increasingly valuable. Equally, as new therapies emerge, defining treatment response in an accurate and timely manner could ensure a rich pipeline of new therapeutic options. International collaboration exists to establish robust data sources for qCT development and, crucially, must ensure that algorithms are widely available once their efficacy has been demonstrated. Research must now pivot to prove the clinical benefit of the widespread adoption of qCT in clinical practice.

**Points for clinical practice**

- qCT has been studied extensively in retrospective patient cohorts with limited prospective validation.
- The role of qCT has been investigated across a range of ILDs including IPF, CTD-ILD and CPFE.
- qCT offers prognostic value even when adjusted for common covariates.
- Serial qCT measurements have shown significant correlation with conventional outcome measures.

**Questions for further research**

- Do qCT biomarkers prospectively align to how patients feel, function and survive?
- Where do qCT biomarkers fit in current clinical guidelines and pathways?
- Can qCT help enhance the pipeline for novel treatments for ILD?
- How do we transition qCT from a promising research tool to a routine clinical outcome measure?
- What is the role of qCT in risk stratifying patients with sub-clinical disease?
- Are qCT markers acceptable and understandable to patients?

## Supplementary material

10.1183/16000617.0194-2024.Supp1 **Please note:** supplementary material is not edited by the Editorial Office, and is uploaded as it has been supplied by the author.
Supplementary information 1: PRISMA checklist ERR-0194-2024.SUPPLEMENT
Supplementary information 2: Search strategies ERR-0194-2024.SUPPLEMENT2
Supplementary table S1 ERR-0194-2024.SUPPLEMENT3
Supplementary table S2 ERR-0194-2024.SUPPLEMENT4
Supplementary table S3 ERR-0194-2024.SUPPLEMENT5
Supplementary table S4 ERR-0194-2024.SUPPLEMENT6
