# Supplementary material for: A systematic review of the role of quantitative CT in the prognostication and disease monitoring of interstitial lung disease
Source: Eur Respir Rev. 2025 Apr 30;34(176):240194. doi: 10.1183/16000617.0194-2024 (PMC12041933; doi:10.1183/16000617.0194-2024)
Supplement: Supplementary file 2 [file ERR-0194-2024.SUPPLEMENT2.pdf]

Supplementary Information 2 Search strategies

CINAHL <1937 to 2024 November 22>

#	Query
S1	(MH "Lung Diseases,Interstitial+")
S2	TI("INTERSTITIAL LUNG") ORAB("INTERSTITIAL LUNG")
S3	TI("idiopathic pulmonary fibrosis")OR AB("idiopathic pulmonaryfibrosis")
S4	TI("interstitial pneumonia*") ORAB("interstitial pneumonia*".
S5	TI("Cryptogenic organizingpneumonia" or "bronchiolitisobliterans organizingpneumonia") OR AB("Cryptogenicorganizing pneumonia" or"bronchiolitis obliteransorganizing pneumonia")
S6	TI("Hamman-Rich syndrome")OR AB("Hamman-Richsyndrome")
S7	TI("Pulmonary sarcoidosis") ORAB ("Pulmonary sarcoidosis")
S8	TI("Hypersensitivity pneumonitis")OR AB("Hypersensitivitypneumonitis")
S9	TI("Pneumoconiosis") ORAB("Pneumoconiosis")
S10	TI("asbestosis") ORAB("asbestosis")
S11	TI("Berylliosis") ORAB("Berylliosis")
S12	TI("Silicosis") OR AB("Silicosis")
S13	TI("Lymphangioleiomyomatosis")ORAB("Lymphangioleiomyomatosis")
S14	TI("Pulmonary Langerhans cellhistiocytosis") OR AB("PulmonaryLangerhans cell histiocytosis")
S15	TI("Birt-Hogg-Dubé syndrome")OR AB("Birt-Hogg-Dubésyndrome")
S16	TI("eosinophilic pneumonia") ORAB("eosinophilic pneumonia")
S17	TI("Radiation pneumonitis") ORAB("Radiation pneumonitis")
S18	TI("Diffuse Parenchymal LungDisease*") OR AB"DiffuseParenchymal Lung Disease*")
S19	TI("allergic alveolitis") ORAB("allergic alveolitis")
S20	TI((("bird fancier*" or farmer*) N1lung*)) OR AB((("bird fancier*" orfarmer*) N1 lung*)))
S21	TI("silo filler*") OR AB("silo filler*")
S22	TI("Trichosporonosis") ORAB("Trichosporonosis")
S23	TI("Anti-Glomerular BasementMembrane") OR AB("Anti-Glomerular BasementMembrane")
S24	TI("Eosinophilic Granuloma") ORAB("Eosinophilic Granuloma")
S25	TI(Anthracosis orAnthracosilicosis) ORAB(Anthracosis orAnthracosilicosis)
S26	TI("Caplan Syndrome") ORAB("Caplan Syndrome")
S27	TI("Siderosis") ORAB("Siderosis")
S28	TI("Silicotuberculosis") ORAB("Silicotuberculosis")
S29	TI("Pulmonary Fibrosis") ORAB("Pulmonary Fibrosis")
S30	TI(Granulomatosis) ORAB(Granulomatosis)
S31	TI("cryptogenic fibrosingalveolitis") OR AB("cryptogenicfibrosing alveolitis")
S32	TI("pleuroparenchymalfibroelastosis") ORAB("pleuroparenchymalfibroelastosis")

S33	S1 OR S2 OR S3 OR S4 OR S5OR S6 OR S7 OR S8 OR S9 ORS10 OR S11 OR S12 OR S13 ORS14 OR S15 OR S16 OR S17OR S18 OR S19 OR S20 ORS21 OR S22 OR S23 OR S24OR S25 OR S26 OR S27 ORS28 OR S29 OR S30 OR S31OR S32
S34	TI((("artificial intelligence" or"machine learn*" or "deep learn*"or "deep-learn*" or "neural net*"or "natural language process*" orradiomic* or quantitative*) and("comput* tomograph*" or"comput* axial tomograph*" or"electron beam tomograph*")))OR AB(("artificial intelligence" or"machine learn*" or "deep learn*"or "deep-learn*" or "neural net*"or "natural language process*" orradiomic* or quantitative*) and("comput* tomograph*" or"comput* axial tomograph*" or"electron beam tomograph*")))
S35	(MH "Artificial Intelligence+") AND(MH "Tomography, X-RayComputed+")
S36	TI("Computer vision") ORAB("Computer vision")
S37	S34 OR S35 OR S36
S38	S33 AND S37
S39	(MH "Vertebrates+") NOT (MH"Human")
S40	S38 NOT S39
S41	(MH "Lung Diseases,Interstitial+")
S42	TI("INTERSTITIAL LUNG") ORAB("INTERSTITIAL LUNG")
S43	TI("idiopathic pulmonary fibrosis")OR AB("idiopathic pulmonaryfibrosis")
S44	TI("interstitial pneumonia*") ORAB("interstitial pneumonia*".
S45	TI("Cryptogenic organizingpneumonia" or "bronchiolitisobliterans organizingpneumonia") OR AB("Cryptogenicorganizing pneumonia" or"bronchiolitis obliteransorganizing pneumonia")
S46	TI("Hamman-Rich syndrome")OR AB("Hamman-Richsyndrome")
S47	TI("Pulmonary sarcoidosis") ORAB ("Pulmonary sarcoidosis")
S48	TI("Hypersensitivity pneumonitis")OR AB("Hypersensitivitypneumonitis")
S49	TI("Pneumoconiosis") ORAB("Pneumoconiosis")
S50	TI("asbestosis") ORAB("asbestosis")
S51	TI("Berylliosis") ORAB("Berylliosis")
S52	TI("Silicosis") OR AB("Silicosis")
S53	TI("Lymphangioleiomyomatosis")ORAB("Lymphangioleiomyomatosis")
S54	TI("Pulmonary Langerhans cellhistiocytosis") OR AB("PulmonaryLangerhans cell histiocytosis")
S55	TI("Birt-Hogg-Dubé syndrome")OR AB("Birt-Hogg-Dubésyndrome")
S56	TI("eosinophilic pneumonia") ORAB("eosinophilic pneumonia")
S57	TI("Radiation pneumonitis") ORAB("Radiation pneumonitis")
S58	TI("Diffuse Parenchymal LungDisease*") OR AB"DiffuseParenchymal Lung Disease*")
S59	TI("allergic alveolitis") ORAB("allergic alveolitis")
S60	TI((("bird fancier*" or farmer*) N1lung*)) OR AB((("bird fancier*" orfarmer*) N1 lung*)))
S61	TI("silo filler*") OR AB("silo filler*")

S62	TI("Trichosporonosis") ORAB("Trichosporonosis")
S63	TI("Anti-Glomerular BasementMembrane") OR AB("Anti-Glomerular BasementMembrane")
S64	TI("Eosinophilic Granuloma") ORAB("Eosinophilic Granuloma")
S65	TI(Anthracosis orAnthracosilicosis) ORAB(Anthracosis orAnthracosilicosis)
S66	TI("Caplan Syndrome") ORAB("Caplan Syndrome")
S67	TI("Siderosis") ORAB("Siderosis")
S68	TI("Silicotuberculosis") ORAB("Silicotuberculosis")
S69	TI("Pulmonary Fibrosis") ORAB("Pulmonary Fibrosis")
S70	TI(Granulomatosis) ORAB(Granulomatosis)
S71	TI("cryptogenic fibrosingalveolitis") OR AB("cryptogenicfibrosing alveolitis")
S72	TI("pleuroparenchymalfibroelastosis") ORAB("pleuroparenchymalfibroelastosis")
S73	S41 OR S42 OR S43 OR S44OR S45 OR S46 OR S47 ORS48 OR S49 OR S50 OR S51OR S52 OR S53 OR S54 ORS55 OR S56 OR S57 OR S58OR S59 OR S60 OR S61 ORS62 OR S63 OR S64 OR S65OR S66 OR S67 OR S68 ORS69 OR S70 OR S71 OR S72
S74	TI((("artificial intelligence" or"machine learn*" or "deep learn*"or "deep-learn*" or "neural net*"or "natural language process*" orradiomic* or quantitative*) and("comput* tomograph*" or"comput* axial tomograph*" or "electron beam tomograph*")))OR AB(("artificial intelligence" or"machine learn*" or "deep learn*"or "deep-learn*" or "neural net*"or "natural language process*" orradiomic* or quantitative*) and("comput* tomograph*" or"comput* axial tomograph*" or"electron beam tomograph*")))
S75	(MH "Artificial Intelligence+") AND(MH "Tomography, X-RayComputed+")
S76	TI("Computer vision") ORAB("Computer vision")
S77	S74 OR S75 OR S76
S78	S73 AND S77
S79	(MH "Vertebrates+") NOT (MH"Human")
S80	S78 NOT S79

Embase <1974 to 2024 November 22>

1 exp interstitial lung disease/
2 "interstitial lung".ti,ab.
3 "idiopathic pulmonary fibrosis".ti,ab.
4 "interstitial pneumonia*".ti,ab.
5 ("Cryptogenic organizing pneumonia" or "bronchiolitis obliterans organizing pneumonia").ti,ab.
6 "Hamman-Rich syndrome".ti,ab.
7 "Pulmonary sarcoidosis".ti,ab.
8 "Hypersensitivity pneumonitis".ti,ab.
9 Pneumoconiosis.ti,ab.
10 asbestosis.ti,ab.
11 Berylliosis.ti,ab.
12 Silicosis.ti,ab.
13 Lymphangioleiomyomatosis.ti,ab.
14 "Pulmonary Langerhans cell histiocytosis".ti,ab.
15 "Birt-Hogg-Dubé syndrome".ti,ab.
16 "eosinophilic pneumonia".ti,ab.
17 "Radiation pneumonitis".ti,ab.
18 "Diffuse Parenchymal Lung Disease*".ti,ab.
19 "allergic alveolitis".ti,ab.
20 (("bird fancier*" or farmer*) adj1 lung*).ti,ab.
21 "silo filler*".ti,ab.
22 Trichosporonosis.ti,ab.
23 "Anti-Glomerular Basement Membrane".ti,ab.
24 "Eosinophilic Granuloma".ti,ab.
25 (Anthracosis or Anthracosilicosis).ti,ab.
26 "Caplan Syndrome".ti,ab.
27 "Siderosis".ti,ab.
28 Silicotuberculosis.ti,ab.
29 "Pulmonary Fibrosis".ti,ab.
30 Granulomatosis.ti,ab.
31 "cryptogenic fibrosing alveolitis".ti,ab.
32 "pleuroparenchymal fibroelastosis".ti,ab.
33 or/1-32
34 (("artificial intelligence" or "machine learn*" or "deep learn*" or "deep-learn*" or "neural net*" or "natural language process*" or radiomic* or quantitative*) and ("comput* tomograph*" or "comput* axial tomograph*" or "electron beam tomograph*")).ti,ab.
35 exp artificial intelligence/ and exp x-ray computed tomography/
36 "Computer vision".ti,ab.
37 or/34-36
38 33 and 37
39 (exp ANIMAL/ or NONHUMAN/) not exp HUMAN/
40 38 not 39
41 40
42 limit 41 to english language

Ovid Emcare <1995 to 2024 Week 47>

1 exp interstitial lung disease/
2 "interstitial lung".ti,ab.
3 "idiopathic pulmonary fibrosis".ti,ab.
4 "interstitial pneumonia*".ti,ab.
5 ("Cryptogenic organizing pneumonia" or "bronchiolitis obliterans organizing pneumonia").ti,ab.
6 "Hamman-Rich syndrome".ti,ab.
7 "Pulmonary sarcoidosis".ti,ab.
8 "Hypersensitivity pneumonitis".ti,ab.
9 Pneumoconiosis.ti,ab.
10 asbestosis.ti,ab.
11 Berylliosis.ti,ab.
12 Silicosis.ti,ab.
13 Lymphangioleiomyomatosis.ti,ab.
14 "Pulmonary Langerhans cell histiocytosis".ti,ab.
15 "Birt-Hogg-Dubé syndrome".ti,ab.
16 "eosinophilic pneumonia".ti,ab.
17 "Radiation pneumonitis".ti,ab.
18 "Diffuse Parenchymal Lung Disease*".ti,ab.
19 "allergic alveolitis".ti,ab.
20 (("bird fancier*" or farmer*) adj1 lung*).ti,ab.
21 "silo filler*".ti,ab.
22 Trichosporonosis.ti,ab.
23 "Anti-Glomerular Basement Membrane".ti,ab.
24 "Eosinophilic Granuloma".ti,ab.
25 (Anthracosis or Anthracosilicosis).ti,ab.
26 "Caplan Syndrome".ti,ab.
27 "Siderosis".ti,ab.
28 Silicotuberculosis.ti,ab.
29 "Pulmonary Fibrosis".ti,ab.
30 Granulomatosis.ti,ab.
31 "cryptogenic fibrosing alveolitis".ti,ab.
32 "pleuroparenchymal fibroelastosis".ti,ab.
33 or/1-32
34 (("artificial intelligence" or "machine learn*" or "deep learn*" or "deep-learn*" or "neural net*" or "natural language process*" or radiomic* or quantitative*) and ("comput* tomograph*" or "comput* axial tomograph*" or "electron beam tomograph*")).ti,ab.
35 exp artificial intelligence/ and exp x-ray computed tomography/
36 "Computer vision".ti,ab.
37 or/34-36
38 33 and 37
39 (exp ANIMAL/ or NONHUMAN/) not exp HUMAN/
40 38 not 39

Ovid MEDLINE(R) ALL <1946 to November 22, 2024>

1 exp Lung Diseases, Interstitial/
2 "interstitial lung".ti,ab.
3 "idiopathic pulmonary fibrosis".ti,ab.
4 "interstitial pneumonia*".ti,ab.
5 ("Cryptogenic organizing pneumonia" or "bronchiolitis obliterans organizing pneumonia").ti,ab.
6 "Hamman-Rich syndrome".ti,ab.
7 "Pulmonary sarcoidosis".ti,ab.
8 "Hypersensitivity pneumonitis".ti,ab.
9 Pneumoconiosis.ti,ab.
10 asbestosis.ti,ab.
11 Berylliosis.ti,ab.
12 Silicosis.ti,ab.
13 Lymphangioleiomyomatosis.ti,ab.
14 "Pulmonary Langerhans cell histiocytosis".ti,ab.
15 "Birt-Hogg-Dubé syndrome".ti,ab.
16 "eosinophilic pneumonia".ti,ab.
17 "Radiation pneumonitis".ti,ab.
18 "Diffuse Parenchymal Lung Disease*".ti,ab.
19 "allergic alveolitis".ti,ab.
20 (("bird fancier*" or farmer*) adj1 lung*).ti,ab.
21 "silo filler*".ti,ab.
22 Trichosporonosis.ti,ab.
23 "Anti-Glomerular Basement Membrane".ti,ab.
24 "Eosinophilic Granuloma".ti,ab.
25 (Anthracosis or Anthracosilicosis).ti,ab.
26 "Caplan Syndrome".ti,ab.
27 "Siderosis".ti,ab.
28 Silicotuberculosis.ti,ab.
29 "Pulmonary Fibrosis".ti,ab.
30 Granulomatosis.ti,ab.
31 "cryptogenic fibrosing alveolitis".ti,ab.
32 "pleuroparenchymal fibroelastosis".ti,ab.
33 or/1-32
34 (("artificial intelligence" or "machine learn*" or "deep learn*" or "deep-learn*" or "neural net*" or "natural language process*" or radiomic* or quantitative*) and ("comput* tomograph*" or "comput* axial tomograph*" or "electron beam tomograph*")).ti,ab.
35 exp Artificial Intelligence/ and exp Tomography, X-Ray Computed/
36 "Computer vision".ti,ab.
37 or/34-36
38 and/33,37
39 exp animals/ NOT exp humans/
40 38 NOT 39
