# Supplementary material for: A systematic review of the role of quantitative CT in the prognostication and disease monitoring of interstitial lung disease
Source: Eur Respir Rev. 2025 Apr 30;34(176):240194. doi: 10.1183/16000617.0194-2024 (PMC12041933; doi:10.1183/16000617.0194-2024)
Supplement: Supplementary file 3 [file ERR-0194-2024.SUPPLEMENT3.pdf]

Supplementary Table S1a Quality Assessment of included journal articles as per Quality in Prognosis Studies (QUIPS) Tool

	Study participation	Study attrition	Prognostic factor measurement	Outcome measurement	Study confounding	Statistical analysis and reporting
Akkaya 2023	Low	Moderate	Low	Low	Low	Low
Aliboni 2022	Moderate	Moderate	High	High	Low	Moderate
Alkhanfar 2022	Moderate	High	Low	High	High	Low
AlNazi 2021	High	High	Moderate	Moderate	Moderate	Moderate
Amorim 2024	Low	Moderate	Low	Low	Low	Low
Ando 2013	Moderate	Moderate	Moderate	Low	Low	Low
Aoki 2022	Low	Low	Low	Low	Moderate	Low
Aoki 2024	Low	Moderate	Low	Low	Low	Low
Argula 2016	Moderate	Moderate	Low	Low	Low	Low
Arzhaeva 2010	Moderate	Low	Low	Moderate	Low	Low
Ash 2017	Low	Moderate	Low	Low	Low	Low
Bak 2019	Low	Low	Low	Low	Low	Low
Barros 2022	Low	Low	Low	Low	Low	Low
Best 2008	Moderate	Moderate	Low	Low	Low	Low
Bocchino 2019	Low	Moderate	Low	Low	Low	Low
Bruni 2022	Low	Low	Low	Low	Low	Low
Budzikowski 2021	Low	High	Low	Low	Moderate	Low
CastilloSaldana 2020	Low	Moderate	Low	Low	Low	Low
Chae 2023	Low	Low	Low	Low	Low	Low
Chassagnon 2021	Moderate	Low	Low	Low	Low	Low
Cheung 2023	Low	Moderate	Low	Low	Low	Low
Choi 2020	Low	Low	Low	Low	Low	Low
Clukers 2018	Low	Moderate	Low	Low	Low	Low

Clukers 2021	Low	Low	Low	Low	Low	Low
Colombi 2015	Low	Moderate	Low	Low	Low	Low
Crews 2020	Low	Low	Low	Low	Low	Low
Devaraj 2024	Low	Moderate	Low	Low	Low	Low
DiBattista 2024	Low	Low	Moderate	Low	Low	Low
Ferrazza 2020	Low	Low	Low	Low	Low	Low
Fukada 2022	Low	Low	Low	Low	Low	Low
Goldin 2018	Low	Moderate	Low	Low	Low	Low
Gudmundsson 2023	Low	Low	Low	Low	Low	Low
Guerra 2023	Low	Low	Low	Low	Low	Low
Handa 2021	Low	Low	Low	Low	Low	Low
Humphries 2017	Low	Low	Low	Low	Low	Low
Humphries 2018	Low	Moderate	Low	Low	Low	Low
Humphries 2022	Low	Low	Low	Low	Low	Low
Humphries 2024	Low	Low	Low	Low	Low	Low
Humphries 2024	Low	Low	Low	Low	Low	Low
Iwasawa 2014	Low	Moderate	Low	Low	Low	Low
Iwasawa 2017	Low	Low	Low	Low	Low	Low
Jacob 2016	Low	Low	Low	Low	Low	Low
Jacob 2017	Low	Low	Low	Low	Low	Low
Jacob 2017	Low	Low	Low	Low	Low	Low
Jacob 2017	Low	Low	Low	Low	Low	Low
Jacob 2017	Low	Low	Low	Low	Low	Low
Jacob 2018	Low	Low	Low	Low	Low	Low
Jacob 2018	Low	Low	Low	Low	Low	Low
Jacob 2019	Low	Low	Low	Low	Low	Low

Jamal 2024	Low	Moderate	Moderate	Low	High	Low
Karampitsakos 2022	Low	High	Low	Low	Low	Low
Khanna 2015	Low	Moderate	Low	Low	Low	Low
Kim 2011	Low	Low	Low	Low	Low	Low
Kim 2015	Low	Low	Low	Low	Low	Low
Kim 2020	Low	Low	Low	Low	Low	Low
Kim 2021	Low	Low	Low	Low	Low	Low
Kim 2021	Low	Low	Low	Low	Low	Low
Ko 2020	Low	Moderate	Low	Low	Low	Low
Koh 2024	Low	Moderate	Low	Low	Low	Low
Koo 2022	Low	Low	Low	Low	Low	Low
Kunihiro 2023	Low	Low	Low	Low	Low	Low
Lancaster 2020	Low	Moderate	Low	Low	Low	Low
Lee 2018	Low	Low	Low	Low	Low	Low
Lee 2021	Low	Low	Low	Low	Low	Low
LeGall 2023	Low	Low	Low	Low	Low	Low
Maetani 2024	Low	Low	Low	Low	Low	Low
Maldonado 2014	Low	Low	Low	Low	Low	Low
Martyanov 2017	Low	Low	Low	Low	Low	Low
Matsuoka 2015	Low	High	Low	Low	Low	Low
Mei 2023	Low	Low	Low	Low	Low	Low
Moon 2016	Low	Low	Low	Low	Low	Low
Moran-Mendoza 2024	Low	Low	Low	Low	Low	Low
Nakagawa 2019	Low	Low	Low	Low	Low	Low
Nam 2023	Low	Low	Low	Low	Low	Low
Nan 2024	Low	Low	Low	Low	Low	Low
Nemoto 2020	Low	Low	Low	Low	Low	Low

Occhipinti 2019	Low	Low	Low	Low	Low	Low
Oh 2023	Low	Low	Low	Low	Low	Low
Oh 2024	Low	Low	Low	Low	Moderate	Low
Ohno 2022	Low	Low	Low	Low	Low	Low
Pan 2022	Low	Low	Low	Low	Low	Low
Park 2023	Low	Low	Low	Low	Low	Low
Qin 2023	Low	Low	Low	Low	Low	Low
Raghu 2016	Low	Low	Low	Low	Low	Low
Robbie 2022	Low	Low	Low	Low	Low	Low
Romei 2020	Low	Moderate	Low	Low	Low	Low
Salaffi 2020	Low	Low	Low	Low	Low	Low
Salhofer 2024	Low	Low	Low	Low	Low	Low
Salisbury 2016	Low	Low	Low	Low	Low	Low
Schniering 2022	Low	Low	Low	Low	Low	Low
Selvan 2024	Low	Low	Low	Low	Low	Low
Shi 2019	Low	Low	Low	Low	Low	Low
Shin 2024	Low	Low	Low	Low	Moderate	Moderate
Shiraishi 2024	Low	Low	Low	Low	Low	Low
Si-Mohamed 2022	Low	Moderate	Low	Low	Low	Low
Steele 2023	Low	Low	Low	Low	Low	Low
Sumikawa 2006	Moderate	Low	Low	Low	Low	Low
Sun 2022	Low	Moderate	Low	Low	Low	Low
Suzuki 2020	Low	Low	Low	Low	Low	Low
Sverzellati 2020	Low	Low	Low	Low	Low	Low
Tanaka 2022	Low	Low	Low	Low	Low	Low
Tanizawa 2015	Low	Low	Low	Low	Low	Low
TemizKaradag 2021	Low	Low	Low	Low	Low	Low

Thillai 2024	Low	Low	Low	Low	Low	Low
Umakoshi 2019	Low	Low	Low	Low	Low	Low
Ungprasert 2017	Low	Moderate	Low	Low	Low	Low
vandenBlink 2016	Low	Low	Low	Low	Low	Low
Venerito 2022	Low	Low	Low	Low	High	Low
Volkmann 2014	Low	Low	Low	Low	Low	Low
Wada 2020	Low	Moderate	Low	Low	Low	Low
Walsh 2022	Low	Low	Low	Low	Low	Low
Wang 2021	Low	Low	Low	Low	Low	Low
Wang 2024	Low	Low	Low	Low	Low	Low
Wong 2021	Low	Low	Low	Low	Low	Low
Xu 2021	Low	Low	Low	Low	Low	Low
Xu 2021	Low	Low	Low	Low	Low	Low
Yamaguchi 2022	Low	Low	Low	Low	Low	Low
Yazawa 2024	Low	Low	Low	Low	Moderate	Moderate
Yoon 2013	Low	Low	Low	Low	Low	Low
Zhao 2023	Low	Low	Low	Low	Low	Low
Zou 2023	Low	Low	Low	Low	Low	Low

Supplementary Table S1b Quality Assessment of included conference abstracts as per Quality in Prognosis Studies (QUIPS) Tool

	Study participation	Study attrition	Prognostic factor measurement	Outcome measurement	Study confounding	Statistical analysis and reporting
Ariani 2015	Moderate	Low	Low	Moderate	Moderate	Low
Ariani 2020	Moderate	Low	Low	Low	High	Moderate
Bae 2020	Low	Low	Low	Low	Low	Low

Belloni 2018	Moderate	High	Moderate	Low	Low	Low
Bosello 2017	Moderate	Low	Low	Low	Moderate	Low
Callahan 2024	Low	Low	Low	Low	Low	Low
Carey 2020	Low	Low	Low	Low	Moderate	Low
Chapman 2023	Low	Low	Low	Low	Low	Low
Chelala 2024	Low	Moderate	Low	Low	Low	Low
DeGiacomi 2018	Low	Low	Low	Low	Low	Low
DeLorenzis 2018	Moderate	Low	Low	Moderate	Moderate	Moderate
Egan 2019	Moderate	Low	Low	Moderate	Low	Moderate
FernandezPerez 2024	Low	Low	Low	Low	Low	Low
George 2023	Low	Low	Low	Low	Low	Low
GodinhoDeAmorim 2023	Low	Low	Low	Low	High	Low
Gorina 2018	Low	Low	Low	Low	Low	Low
Hinze 2021	Low	Low	Low	Low	Low	Low
Hinze 2021	Low	Low	Low	Low	Low	Low
Humphries 2017	Low	Low	Low	Low	Low	Low
Humphries 2021	Low	Low	Low	Low	Low	Low
Humphries 2021	Low	Low	Low	Low	Low	Low
Humphries 2024	Low	Low	Low	Moderate	Low	Low
HyunOh 2021	Moderate	Low	Low	Low	Low	Low
Jacob 2017	Low	Low	Low	Low	Low	Low
Jacob 2019	Low	Low	Low	Low	Low	Low
Jo 2020	Low	Low	Low	Low	Low	Low
Jo 2020	Low	Low	Low	Low	Low	Low
Kim 2015	Low	Low	Low	Low	Low	Low
Kim 2016	Low	Low	Low	Low	Low	Low
Kim 2017	Low	Low	Low	Low	Low	Low

Kim 2018	Low	Low	Low	Low	Low	Low
Kim 2018	Low	Low	Low	Low	Moderate	Low
Kim 2019	Low	Low	Low	Low	Low	Low
Kim 2021	Low	Low	Low	Low	Low	Low
Kim 2022	Low	Low	Low	Low	Low	Low
Kim 2023	Low	Low	Low	Low	Low	Low
Kim 2023	Low	Low	Low	Low	Low	Low
Kirov 2023	Low	Low	Low	Low	Low	Low
Kitaguchi 2023	Low	Low	Low	Low	Low	Low
Koslow 2023	Low	Low	Low	Low	Low	Low
Koslow 2024	Low	Moderate	Low	Low	Moderate	Low
Kreuter 2018	Low	Low	Low	Low	Low	Low
Loh 2019	Low	Low	Low	Low	Low	Low
Matson 2024	Low	Low	Low	Low	Low	Low
Moua 2011	Low	Low	Low	Low	Low	Low
Mutha 2024	Low	Low	Low	Low	Moderate	Low
Nakagawa 2017	Low	Low	Low	Low	Low	Low
Nakagawa 2018	Low	Low	Low	Low	Low	Low
Nakagawa 2019	Low	Low	Low	Low	Low	Low
Putman 2024	Low	Low	Low	Low	Low	Low
Roberts 2011	Moderate	Low	Low	Moderate	Low	Low
Roberts 2021	Low	Low	Low	Low	Low	Low
Selvan 2024	Low	Low	Low	Low	Low	Low
Simha 2024	Low	Low	Low	Low	Low	Low
Song 2019	Low	Low	Low	Low	Low	Low
Suzuki 2024	Low	Low	Low	Low	Low	Low
Thillai 2022	Low	Low	Low	Low	Low	Low

Ussavarungsi 2023	Low	Low	Low	Low	Low	Low
Vettori 2018	Low	Low	Low	Low	Low	Low
Walsh 2024	Low	Low	Low	Low	Low	Low
Wang 2024	Low	Low	Low	Low	Low	Low
Yagihashi 2015	Low	Low	Low	Low	Low	Low
Yeo 2022	Low	Low	Low	Low	Low	Low
YingyingFang 2023	Low	Low	Low	Low	Low	Low
