# Supplementary material for: A systematic review of the role of quantitative CT in the prognostication and disease monitoring of interstitial lung disease
Source: Eur Respir Rev. 2025 Apr 30;34(176):240194. doi: 10.1183/16000617.0194-2024 (PMC12041933; doi:10.1183/16000617.0194-2024)
Supplement: Supplementary file 4 [file ERR-0194-2024.SUPPLEMENT4.pdf]

Supplementary Table S2 Summary data for journal articles where primary interstitial lung disease subtype is idiopathic pulmonary fibrosis

Author	Year	Study design	Prognostication	Disease monitoring	Total number of participants included in analysis	Quantitative CT modality	Quantitative CT feature	Prognostication			Disease monitoring		
								Reported outcome statistic	Duration of follow up (Median unless otherwise specified)	Multivariate adjustments	Reported outcome statistic	Interval	Correlation
Akkaya *et al*[1]	2023	Retrospective study (Proprietary)	✓	X	57	Histogram using Phillips IntelliSpace	Mediastinal adipose tissue	Risk of progression OR 0.991 (0.984-0.997)<br>Mortality OR 0.993 (0.975-1.011)	"12 months"	n/a			
Al Nazi *et al*[2]	2021	Retrospective study (Online resource)	✓	X	176	Fibro-CoSANet	CNN combining CT and clinical data	Risk of progression Laplace Log-likelihood score-6.68	"1-2 years"	n/a			
Ash *et al*[3]	2017	Retrospective study (Proprietary)	✓	X	46	Histogram and radiographic feature subtype	Histogram analysis	Univariate analysis: Death or transplant Kurtosis: HR 4.49, p=0.001	465 days	Unadjusted			
Bak *et al*[4]	2019	Retrospective study (Proprietary)	✓	X	205	VisualC++ and Insight Segmentation and Registration Toolkit	Consensus hierarchical cluster analysis of clinical features and texture-based automated quantification	Significant differences in survival between clusters (p=0.019)	Not given	n/a			
Best *et al*[5]	2008	Secondary analysis of prospective study	✓	X	167	Histogram	Kurtosis and fibrosis extent	Multivariate logistic regression analyses for mortality prediction<br>Kurtosis OR 0.579 (0.32-1.049), p=0.072<br>Fibrosis OR 1.104 (1.018-1.198), p=0.017	1.5 years	FVC, TLC, visual CT score			
Budzikowski *et al*[6]	2021	Secondary analysis of retrospective study	✓	X	169	Matlab- first-order histogram, gray-level co-occurrence matrix, fractal, Fourier, and Laws filter features	142 radiomic features;<br>First-order histogram, gray-level co-occurrence matrix, fractal, Fourier and Laws' filter features combined with known genetic mutations	Two first order histogram features (Mean and Thresh95) demonstrated best discrimination between patient survival curves. Both metrics p<0.001	Not given	n/a			

Cheung *et al[7]*	2023	Retrospective study (Proprietary)	✓	X	90	AirQuant	Intersegmental tapering<br>Segmental tortuosity	Multivariate analysis for mortality<br>Adjusted for FVC:<br>Intersegmental tapering HR 0.749 (0.664-0.845) $p<0.001$<br>Segmental tortuosity HR 1.736 (1.222-2.466) $p<0.002$	2.68 years	FVC			
Clukers *et a[8]l*	2018	Secondary analysis of prospective study	X	✓	89	Functional respiratory imaging	Lung volumes, specific image based airway radius (siRADaw)				Mixed-effect model comparing a) ΔFVC vs Δlung volumes<br>b) ΔFVC vs ΔsiRADaw	48 weeks	a) $R^2=0.18$, $p<0.001$,<br>b) $R^2=0.15$, $p=0.002$
Colombi *et al[9]*	2015	Retrospective study (Proprietary)	X	✓	40	Lung density histogram	Lung density				Pearson correlation coefficient<br>a) $\Delta 40^{th}$ and $\Delta 80^{th}$ percentile lung density<br>b) $\Delta 40^{th}$ vs overall visual progression	Median 13 months	a) $r^2=0.69$, $p<0.001$<br>b) $r^2 =0.58$, $p < 0.001$
Devaraj *et al[10]*	2024	Secondary analysis of prospective study	✓	X	62	e-LUNG	Weighted reticulovascular score	WRVS, age and FVC associated with FVC decline C-index 0.81	52 weeks	N/A			
Handa *et al[11]*	2022	Primary prospective study	✓	X	120	AIQCT	Bronchial volumes<br>Normal lung volumes	Multivariate analysis for survival<br>Bronchial volumes HR 1.33 (1.16-1.53)<br>Normal lung volumes HR 0.97 (0.94-0.99)	2184 days (5.98 years)	GAP score			
Humphries *et al[12]*	2017	Secondary analysis of prospective study	X	✓	335	DTA	DTA score				Spearman correlation coefficients<br>a) ΔDTA score vs ΔFVC%<br>b) ΔDTA score vs ΔFVC%	Median 14.7 months	a) $r=-0.41$, $p<0.001$<br>b) $r=-0.40$, $p<0.001$
Humphries *et al[13]*	2018	Secondary analysis of prospective study	X	✓	141	DTA	DTA score				Spearman correlation coefficients<br>a) ΔDTA score vs ΔFVC%<br>b) ΔDTA score vs ΔDLCO%<br>c) ΔDTA score vs Δ6MWD%	12.5 or 15 months	a) $r=-0.46$, $p<0.001$<br>b) $r=-0.38$, $p<0.001$<br>c) $r=-0.30$, $p<0.001$<br>d) $r=-0.37$, $p<0.001$

											d) ΔDTA score vs ΔSGRQ%		
Humphries *et al[14]*	2022	Secondary analysis of prospective study	✓	X	393	DTA	DTA	Cox proportional hazards models Transplant free survival HR 1.2 (1.14-1.26) Progression free survival HR 1.14 (1.08-1.19)	Median 2.7 years	Age, Sex, BMI, smoking history and anti-fibrotic therapy			
Iwasawa *et al[15]*	2014	Retrospective study (Proprietary)	X	✓	78	Gaussian Histogram Normalized Correlation	Fibrosis lesion				Pearsons rank correlation coefficient ΔF-pattern volume vs ΔVC	Mean 12.8 months	r=-0.572, p<0.001
Jacob *et al[16]*	2017	Retrospective study (Proprietary)	✓	X	283	CALIPER	PVV, Honeycombing %	Multivariate cox regression analysis for mortality PVV HR 1.53 (1.41-1.66) p<0.0001 Honeycombing HR 1.12 (1.04-1.21) p=0.004	Mean 30.5	Pulmonary function testing			
Jacob *et al[17]*	2018	Retrospective study (Proprietary)	✓	X	363	CALIPER	VRS (PVV) Upper zone (UZ) VRS	Total VRS and upper zone VRS predicted survival	Not given	Sex, age at CT imaging, smoking status			
Jacob *et al[18]*	2018	Retrospective study (Proprietary)	X	✓	66	CALIPER	PVV				Univariate linear regression analysis a) ΔTotal ILD extent vs ΔFVC% b) ΔTotal PVV extent vs ΔFVC%	Mean 1.1 years	a) r^2=0.53, p<0 .0001 b) r^2=0.57, p<0 .0001
Kim *et al[19]*	2015	Retrospective study (Proprietary)	X	✓	57	QLF, Histogram	QILD QLF volume Histogram kurtosis				Spearman rank correlations a) ΔQLF score vs ΔFVC% b) ΔQLF score vs ΔDLCO% c) Kurtosis vsΔFVC%	Mean 7 months	a) r=-0.57, p<0.001 b) r=-0.49, p=0.001 c) r=0.26, p<0.0513
Kim *et al[20]*	2020	Secondary analysis of prospective study	✓	X	192	QLF	QLF	Landmark cox regressions for risk of progression QLF score in worst affected lobe associated with higher risk of progression HR 5.92 (SE 3.11), p=0.001	Pilot 18 months Validation median 19 months after 6/12 landmark	GAP score			

Kim *et al*[21]	2021	Retrospective study (Proprietary)	✓	X	193	QLF; STP score	STP score	Cox proportional hazard regressions for prediction of progression free survival defined by 10% loss in FVC or 4% progression of QLF score:<br>4% QLF progression HR 1.53, p=0.041<br>10% FVC progression HR 0.92, p=0.70	Mean 7.6 months HRCT an 8.0 months PFTs	Age<br>Sex			
Kim *et al*[22]	2021	Secondary analysis of prospective study	X	✓	137	QLF	QLF whole lung<br>QILD whole lung				Spearman rank correlations<br>a) ΔQLF score % vs ΔFVC%<br>b) ΔQLF score % vs ΔDLCO%<br>c) ΔQLF score % vs ΔUSCD-SOBQ score %<br>d) ΔQILD score % vs ΔFVC%<br>e) ΔQILD score % vs ΔDLCO%<br>f) ΔQILD score % vs ΔUSCD-SOBQ score %	26 weeks	a) r=-0.4142, p<0.0001<br>b) r=-0.2166, p=0.0196<br>c) r=-0.2680, p=0.0034<br>d) r=-0.3775, p<0.0001<br>e) r=-0.2136, p=0.0213<br>f) r=-0.1972, p=0.0323
Koo *et al*[23]	2022	Primary prospective study	X	✓	38	CALIPER	VRS (PVV)<br>ILD				Repeated measure correlation coefficient<br>a) ΔVRS % vs ΔFVC% p<0.0001<br>b) ΔILD % vs ΔFVC%	12 months	a)-0.491, (-0.685--0.251)<br>b)-0.343, (-0.547--0.108)<br>p<0.004
Kunihiro *et al*[24]	2023	Retrospective study (Proprietary)	✓	✓	48	GHNC	GHNC score for GGA and honeycombing	Multivariate Cox regression analysis for survival<br>GGA HR 0.85 (0.72-0.99), p=0.0384<br>Honeycombing HR 1.40 (1.03-1.89) p=0.0314	Median 1190 days	Age, sex, presence of therapies for IPF, thoracic surgery history, lung cancer presence, serum KL-6 levels, %VC values at the timing of initial CT examinations, and GHNC	Spearmans correlation analysis between progression of IPF per year and GHNC scores of<br>a) honeycombing<br>b) reticulation<br>c) consolidation	Median 1190 days	a) r=0.4649<br>b) r=0.4128<br>c) r=0.4375

Lancaster *et al* [25]	2020	Primary prospective study	X	✓	113	QLF	QLF score (%)<br>QLF score (ml)				Pearson and Spearman correlation coefficients<br>a) ΔQLF score (%) % vs ΔFVC%<br>b) ΔQLF score (ml) % vs ΔFVC%	6 months	a) r^2=0.34<br>b) r^2=0.43
Lee *et al[26]*	2018	Retrospective study (Proprietary)	✓	X	144	Fibrosis score (sum of extent of honeycombing and reticular opacities)	Fibrosis score<br>Interval change in fibrosis score	Multivariate Cox hazard analysis for survival<br>Fibrosis score HR 1.033 (1.014-1.052), p=0.001)<br>Interval change in fibrosis score HR 1.049 (1.026-1.072), p<0.001	Median 57.9 months	Age, FVC, DLCO, 6MWD and desaturation			
Maetani *et al[27]*	2024	Retrospective study (Proprietary)	✓	X	106	SYNAPSE VINCENT	Wall area of segmental and subsegmental airways (WAintra)	WAintra associated with mortality across 4 models HR 1.58-1.63 (CI 1.11-2.34) (p<0.001)	10 years	Age, height, smoking, FVC, DLCO, %fibrosis, normal lung volume, airway volume			
Maldonado *et al[28]*	2014	Retrospective study (Proprietary)	✓	X	55	CALIPER	Change in %ILD, total ILD volume, total reticular volume at mean 289 days	Cox proportional hazards regression survival analysis<br>Change in percentage ILD HR 1.52 (1.08-2.15) p<0.017<br>Total ILD volume HR 1.70, (1.19-2.43) p<0.003<br>Total reticulation volume HR 1.91, (1.21-3.0) p<0.006	Median 2.4 years	Sex, smoking pack-years, FVC, DLCO, interval between CTs			
Nakagawa *et al[29]*	2019	Retrospective study (Proprietary)	✓	X	52	Honeycomb area	Percentage of HA to total lung area (%HA)	Multivariable logistic regression analysis for survival<br>%HA OR 1.27, (1.05-1.62), p=0.011	Median 3.4 years	Age, Sex, BMI, pack-years			
Nam *et al[30]*	2023	Retrospective study (Proprietary)	✓	X	161	AVIEW Deep learning software	Normal Lung Volume Proportion and Fibrotic lung volume proportion	Multivariable Cox proportional hazard models for survival<br>Normal Lung HR 0.97 (0.96-0.98) p<0.001<br>Fibrotic lung 1.03 (1.01-1.05) p=0.007	Median 54.1 months	Age, Sex, Comorbidities, smoking status, FVC and DLCO			

Pan *et al[31]*	2023	Retrospective study (Proprietary)	✓	X	92	Unsupervised machine learning	Radiological disease progression signatures	Kaplan-Meier comparison of clustering of disease progression signatures and interval disease progression signatures identified significantly different survival outcomes	Not given	n/a			
Raghu *et al[32]*	2016	Primary prospective study	X	✓	89	QLF	QLF<br>GG<br>QILD				Pearson correlation coefficient<br>a) ΔQLF score (%) % vs ΔFVC% (24 weeks)<br>b) ΔQLF score (%) % vs ΔFVC% (48 weeks)<br>c) ΔGG score (%) % vs ΔFVC% (48 weeks)<br>d) ΔQILD score (%) % vs ΔFVC% (48 weeks)	24 and 48 weeks	a) r=-0.520; p<0.0001<br>b) r=-0.624 p<0.001<br>c) r=-0.233, p=0.074<br>d) r=-0.514, p<0.001
Robbie *et a[33]l*	2022	Retrospective study (Proprietary)	✓	✓	81	Syngo CT Pulmo3D package	Automated lung volume	Multivariate regression analysis for mortality<br>Annual change in ALV HR 0.98, p<0.021	Median 12 months	Baseline disease severity, age, and sex	Linear regression analysis of ΔALV vs ΔFVC	Median 12 months	R^2 = 0.26, p<0.0001
Romei *et a[34]l*	2020	Retrospective study (Proprietary)	✓	✓	44	CALIPER	ILD %<br>PVV %	Unpaired t-test of “Slow decliners” vs “Fast decliners” showed difference in baseline ILD%, 15.0±1.9 vs. 22.7±3.3;and PVV% 4.3±0.2vs.5.2±0.4	36 months	n/a	Linear correlation coefficients of CALIPER measures (a) ILD% and b) PVV%) vs FVC	Not given	a) r^2 = 0.35 p<0.0001<br>b) r^2 = 0.19 p= 0.0031
Salisbury *et al[35]*	2016	Primary prospective study	✓	✓	199	Adaptive Multiple Features Method	Ground glass reticular score	Multivariable Cox Proportional Hazards Models for association with disease progression HR 1.35 (1.01-1.76) p=0.04	60 weeks	GAP score, smoking status	Linear correlation coefficient of AMFM measured GGR and FVC	60 weeks	r=-0.25, (-0.42--0.06, p=0.01
Shi *et al[36]*	2019	Retrospective study (Proprietary)	✓	X	50	QPSO-RF algorithm	19 texture features	19 texture features to predict radiological progression in ROI. Sensitivity 81.8%, specificity 82.2%, Accuracy 82.1%	Median 7.3 months	n/a			

Sun *et al[37]*	2022	Retrospective study (Proprietary)	X	✓	69	Elastic registration technique (ElasticSyN)	Mean log_jac value				a) Δmean log_jac (%) % vs ΔVC% b) Δmean log_jac (%) % vs ΔFVC%	Median 13.1 months	a) r=0.394 p<0.01 b) r=0.395 p<0.05
Sverzellati *et al[38]*	2020	Retrospective study (Proprietary)	✓	X	58	CALIPER	>20% increase in total lung fibrosis	Adjusted Cox proportional hazard models for survival HR 2.89 (1.13-8.37) Combined with >10% drop in FVC% HR 12.1 (3.1-46.7)	Median 44 months	GAP score, pack year history			
Tanaka *et al[39]*	2022	Retrospective study (Proprietary)	✓	X	140	3D-CT Lung Volume	Standardised 3D-CT lung volume %	Multivariable Cox proportional hazard model for survival HR 0.978, (0.946-0.991) p=0.002	3.7 years	Age, Sex			
Thillai *et al[40]*	2024	Retrospective study (Proprietary)	✓	X	446	Fibrosis and airway models	Fibrosis volume	Fibrosis volume independently associated with reduced 2 year PFS HR 1.17 (1.12-1.22) p<0.001	39.1 months	GAP score			
Van den Blink *et al[41]*	2016	Secondary analysis of prospective study	X	✓	21	CALIPER	Non-ILA % volume				No correlation between ΔFVC % and non-ILA %	57 days	Not shown
Walsh *et a[42]l*	2022	Retrospective study (Proprietary)	✓	X	504	SOFIA	SOFIA PIOPED UIP probability	Adjusted Cox proportional hazards modelling for transplant free survival HR 1.29 (1.17-1.41) p<0.0001	Not given	Age, Sex, Total ILD extent, FVC, DLCO, CPI, GAP			
Wang *et al[43]*	2024	Primary prospective study	✓	X	102	Aview	Honeycombing	OR 1.081 (1.02-1.143) p=0.013 per 1% honeycombing for AE-IPF					
Wong *et al[44]*	2021	Retrospective study (Online resource)	✓	X	200	Fibrosis-NET	Fibrosis-NET score	Modified Laplace Log Likelihood score for prediction of progression -6.8188	"1-3 years"	n/a			

AIQCT = Artificial intelligence-based quantitative CT image analysis software, AE-IPF = acute exacerbation of idiopathic pulmonary fibrosis, ALV = automated lung volume, AMFM = Adaptive Multiple Features Method, BMI = Body mass index, CALIPER = Computer-Aided Lung Informatics for Pathology Evaluation and Rating, CNN = Convolutional neural network, CPFE = Combined pulmonary fibrosis and emphysema, CPI = Composite physiological index, CT = Computed tomography, DLCO = Diffusion capacity of lung for carbon monoxide, DTA = Data driven textual analysis, FVC = Forced vital capacity, GAP = Gender Age Physiology, GGA = ground glass area, GGR = ground glass reticular, GHNC = Gaussian histogram normalized correlation, HA=Honeycomb area, HR = Hazard ratio, ILA = Interstitial lung abnormality, ILD = Interstitial lung disease, IPF = Idiopathic pulmonary fibrosis, OR = Odds ratio, PFS = progression free survival, PVV = pulmonary vessel volume, QILD = Quantitative Interstitial Lung Disease, QLF = Quantitative Lung Fibrosis, QPSO-RF = quantum particle swarm optimization-random forest, ROI = region of interest, siRADaw = Specific image based airway radius, SOFIA =

Systematic Objective Fibrotic Imaging Analysis Algorithm, STP = Single scan probability score, TLC = Total lung capacity, USCD-SOBQ = University of California San Diego Shortness of Breath Questionnaire, UZ = upper zone, VC = vital capacity, VRS = Vessel related structures, 6MWD = 6 minute walk distance

1. Akkaya H, Ercen Diken O. Can lung semi-quantitative measurements and mediastinal adipose tissue volume predict prognosis in patients with idiopathic pulmonary fibrosis (IPF)? A CT-based preliminary study. *Tuberk Toraks* 2023: 71(3): 203-214.
2. Al Nazi Z, Rabbi Mashrur F, Islam MA, Saha S. Fibro-CoSANet: pulmonary fibrosis prognosis prediction using a convolutional self attention network. *Physics in medicine and biology* 2021: 66(22).
3. Ash SY, Harmouche R, Lopez Vallejo DL, Villalba JA, Ostridge K, Gunville R, Come CE, Onieva JO, Ross JC, Hunninghake GM, El-Chemaly SY, Doyle TJ, Nardelli P, Sanchez-Ferrero GV, Goldberg HJ, Rosas IO, Jose Estepar RS, Washko GR, Vallejo DLL, Onieva Onieva J. Densitometric and local histogram based analysis of computed tomography images in patients with idiopathic pulmonary fibrosis. *Respiratory Research* 2017: 18: 1-11.
4. Bak SH, Park HY, Nam JH, Lee HY, Lee JH, Sohn I, Chung MP. Predicting clinical outcome with phenotypic clusters using quantitative CT fibrosis and emphysema features in patients with idiopathic pulmonary fibrosis. *PLoS One* 2019: 14(4): e0215303.
5. Best AC, Meng J, Lynch AM, Bozic CM, Miller D, Grunwald GK, Lynch DA. Idiopathic pulmonary fibrosis: physiologic tests, quantitative CT indexes, and CT visual scores as predictors of mortality. *Radiology* 2008: 246(3): 935-940.
6. Budzikowski JD, Foy JJ, Rashid AA, Chung JH, Noth I, Armato SG, 3rd. Radiomics-based assessment of idiopathic pulmonary fibrosis is associated with genetic mutations and patient survival. *J Med Imaging (Bellingham)* 2021: 8(3): 031903.
7. Cheung WK, Pakzad A, Mogulkoc N, Needleman S, Rangelov B, Gudmundsson E, Zhao A, Abbas M, McLaverty D, Asimakopoulos D, Chapman R, Savas R, Janes SM, Hu Y, Alexander DC, Hurst JR, Jacob J. Automated airway quantification associates with mortality in idiopathic pulmonary fibrosis. *Eur Radiol* 2023: 33(11): 8228-8238.
8. Clukers J, Lanclus M, Mignot B, Van Holsbeke C, Roseman J, Porter S, Gorina E, Kouchakji E, Lipson KE, De Backer W, De Backer J. Quantitative CT analysis using functional imaging is superior in describing disease progression in idiopathic pulmonary fibrosis compared to forced vital capacity. *Respir Res* 2018: 19(1): 213.
9. Colombi D, Dinkel J, Weinheimer O, Obermayer B, Buzan T, Nabers D, Bauer C, Oltmanns U, Palmowski K, Herth F, Kauczor HU, Sverzellati N, Kreuter M, Heussel CP. Visual vs Fully Automatic Histogram-Based Assessment of Idiopathic Pulmonary Fibrosis (IPF) Progression Using Sequential Multidetector Computed Tomography (MDCT). *PLoS One* 2015: 10(6): e0130653.
10. Devaraj A, Ottink F, Rennison-Jones C, Blé F-X, Joly O, Azim A, Gerry S, Harston G, Ostridge K, George PM. e-Lung Computed Tomography Biomarker Stratifies Patients at Risk of Idiopathic Pulmonary Fibrosis Progression in a 52-Week Clinical Trial. *American Journal of Respiratory and Critical Care Medicine* 2024: 209(9): 1168-1169.
11. Handa T, Tanizawa K, Oguma T, Uozumi R, Watanabe K, Tanabe N, Niwamoto T, Shima H, Mori R, Nobashi TW, Sakamoto R, Kubo T, Kurosaki A, Kishi K, Nakamoto Y, Hirai T. Novel Artificial Intelligence-based Technology for Chest Computed Tomography Analysis of Idiopathic Pulmonary Fibrosis. *Ann Am Thorac Soc* 2022: 19(3): 399-406.
12. Humphries SM, Yagihashi K, Huckleberry J, Rho BH, Schroeder JD, Strand M, Schwarz MI, Flaherty KR, Kazerooni EA, van Beek EJR, Lynch DA. Idiopathic Pulmonary Fibrosis: Data-driven Textural Analysis of Extent of Fibrosis at Baseline and 15-Month Follow-up. *Radiology* 2017: 285(1): 270-278.
13. Humphries SM, Swigris JJ, Brown KK, Strand M, Gong Q, Sundy JS, Raghu G, Schwarz MI, Flaherty KR, Sood R, O'Riordan TG, Lynch DA. Quantitative high-resolution computed tomography fibrosis score: performance characteristics in idiopathic pulmonary fibrosis. *Eur Respir J* 2018: 52(3).
14. Humphries SM, Mackintosh JA, Jo HE, Walsh SLF, Silva M, Calandriello L, Chapman S, Ellis S, Glaspole I, Goh N, Grainge C, Hopkins PMA, Keir GJ, Moodley Y, Reynolds PN, Walters EH, Baraghoshi D, Wells AU, Lynch DA, Corte TJ. Quantitative computed tomography predicts outcomes in idiopathic pulmonary fibrosis. *Respirology* 2022: 27(12): 1045-1053.
15. Iwasawa T, Ogura T, Sakai F, Kanauchi T, Komagata T, Baba T, Gotoh T, Morita S, Yazawa T, Inoue T. CT analysis of the effect of pirfenidone in patients with idiopathic pulmonary fibrosis. *Eur J Radiol* 2014: 83(1): 32-38.
16. Jacob J, Bartholmai BJ, Rajagopalan S, Kokosi M, Nair A, Karwoski R, Walsh SLF, Wells AU, Hansell DM. Mortality prediction in idiopathic pulmonary fibrosis: evaluation of computer-based CT analysis with conventional severity measures. *The European respiratory journal* 2017: 49(1).
17. Jacob J, Bartholmai BJ, Rajagopalan S, van Moorsel CHM, van Es HW, van Beek FT, Struik MHL, Kokosi M, Egashira R, Brun AL, Nair A, Walsh SLF, Cross G, Barnett J, de Lauretis A, Judge EP, Desai S, Karwoski R, Ourselin S, Renzoni E, Maher TM, Altmann A, Wells AU. Predicting Outcomes in Idiopathic Pulmonary Fibrosis Using Automated Computed Tomographic Analysis. *Am J Respir Crit Care Med* 2018: 198(6): 767-776.
18. Jacob J, Bartholmai BJ, Rajagopalan S, Kokosi M, Egashira R, Brun AL, Nair A, Walsh SLF, Karwoski R, Wells AU. Serial automated quantitative CT analysis in idiopathic pulmonary fibrosis: functional correlations and comparison with changes in visual CT scores. *Eur Radiol* 2018: 28(3): 1318-1327.
19. Kim HJ, Brown MS, Chong D, Gjertson DW, Lu P, Kim HJ, Coy H, Goldin JG. Comparison of the quantitative CT imaging biomarkers of idiopathic pulmonary fibrosis at baseline and early change with an interval of 7 months. *Acad Radiol* 2015: 22(1): 70-80.
20. Kim GHJ, Weigt SS, Belperio JA, Brown MS, Shi Y, Lai JH, Goldin JG. Prediction of idiopathic pulmonary fibrosis progression using early quantitative changes on CT imaging for a short term of clinical 18-24-month follow-ups. *Eur Radiol* 2020: 30(2): 726-734.
21. Kim GHJ, Shi Y, Yu W, Wong WK. A study design for statistical learning technique to predict radiological progression with an application of idiopathic pulmonary fibrosis using chest CT images. *Contemp Clin Trials* 2021: 104: 106333.
22. Kim GHJ, Goldin JG, Hayes W, Oh A, Soule B, Du S. The value of imaging and clinical outcomes in a phase II clinical trial of a lysophosphatidic acid receptor antagonist in idiopathic pulmonary fibrosis. *Ther Adv Respir Dis* 2021: 15: 17534666211004238.
23. Koo CW, Larson NB, Parris-Skeete CT, Karwoski RA, Kalra S, Bartholmai BJ, Carmona EM. Prospective machine learning CT quantitative evaluation of idiopathic pulmonary fibrosis in patients undergoing anti-fibrotic treatment using low- and ultra-low-dose CT. *Clin Radiol* 2022: 77(3): e208-e214.
24. Kunihiro Y, Matsumoto T, Murakami T, Shimokawa M, Kamei H, Tanaka N, Ito K. A quantitative analysis of long-term follow-up computed tomography of idiopathic pulmonary fibrosis: the correlation with the progression and prognosis. *Acta Radiol* 2023: 64(8): 2409-2415.
25. Lancaster L, Goldin J, Trampisch M, Kim GH, Ilowite J, Homik L, Hotchkin DL, Kaye M, Ryerson CJ, Mogulkoc N, Conoscenti CS. Effects of Nintedanib on Quantitative Lung Fibrosis Score in Idiopathic Pulmonary Fibrosis. *Open Respir Med J* 2020: 14: 22-31.
26. Lee SM, Seo JB, Oh SY, Kim TH, Song JW, Kim N. Prediction of survival by texture-based automated quantitative assessment of regional disease patterns on CT in idiopathic pulmonary fibrosis. *European Radiology* 2018: 28(3): 1293-1300.

27. Maetani T, Tanabe N, Tanizawa K, Sakamoto R, Shiraishi Y, Hayashi Y, Uyama M, Matsunashi A, Sato S, Suzuki K, Masuda I, Fukui M, Kaji S, Handa T, Hirai T. Computed tomography morphological assessments of central airways in interstitial lung abnormalities and idiopathic pulmonary fibrosis. *Respiratory research* 2024: 25(1): 404.
28. Maldonado F, Moua T, Rajagopalan S, Karwoski RA, Raghunath S, Decker PA, Hartman TE, Bartholmai BJ, Robb RA, Ryu JH. Automated quantification of radiological patterns predicts survival in idiopathic pulmonary fibrosis. *Eur Respir J* 2014: 43(1): 204-212.
29. Nakagawa H, Ogawa E, Fukunaga K, Kinose D, Yamaguchi M, Nagao T, Tanaka-Mizuno S, Nakano Y. Quantitative CT analysis of honeycombing area predicts mortality in idiopathic pulmonary fibrosis with definite usual interstitial pneumonia pattern: A retrospective cohort study. *PLoS One* 2019: 14(3): e0214278.
30. Nam JG, Choi Y, Lee S-M, Yoon SH, Goo JM, Kim H. Prognostic value of deep learning-based fibrosis quantification on chest CT in idiopathic pulmonary fibrosis. *European radiology* 2023: 33(5): 3144-3155.
31. Pan J, Hofmanninger J, Nenning K-H, Prayer F, Rohrich S, Sverzellati N, Poletti V, Tomassetti S, Weber M, Prosch H, Langs G. Unsupervised machine learning identifies predictive progression markers of IPF. *European radiology* 2022(cl3, 9114774).
32. Raghu G, Scholand MB, de Andrade J, Lancaster L, Mageto Y, Goldin J, Brown KK, Flaherty KR, Wencel M, Wanger J, Neff T, Valone F, Stauffer J, Porter S. FG-3019 anti-connective tissue growth factor monoclonal antibody: results of an open-label clinical trial in idiopathic pulmonary fibrosis. *Eur Respir J* 2016: 47(5): 1481-1491.
33. Robbie H, Wells AU, Fang C, Jacob J, Walsh SLF, Nair A, Camoras R, Desai SR, Devaraj A. Serial decline in lung volume parameters on computed tomography (CT) predicts outcome in idiopathic pulmonary fibrosis (IPF). *Eur Radiol* 2022: 32(4): 2650-2660.
34. Romei C, Tavanti LM, Taliani A, De Liperi A, Karwoski R, Celi A, Palla A, Bartholmai BJ, Falaschi F. Automated Computed Tomography analysis in the assessment of Idiopathic Pulmonary Fibrosis severity and progression. *Eur J Radiol* 2020: 124: 108852.
35. Salisbury ML, Lynch DA, van Beek EJR, Kazerooni EA, Guo J, Xia M, Murray S, Anstrom KJ, Yow E, Martinez FJ, Hoffman EA, Flaherty KR. Idiopathic Pulmonary Fibrosis: The Association between the Adaptive Multiple Features Method and Fibrosis Outcomes. *American Journal of Respiratory and Critical Care Medicine* 2016: 195(7): 921-929.
36. Shi Y, Wong WK, Goldin JG, Brown MS, Kim GHJ. Prediction of progression in idiopathic pulmonary fibrosis using CT scans at baseline: A quantum particle swarm optimization - Random forest approach. *Artif Intell Med* 2019: 100: 101709.
37. Sun H, Yang X, Sun X, Meng X, Kang H, Zhang R, Zhang H, Liu M, Dai H, Wang C. Lung shrinking assessment on HRCT with elastic registration technique for monitoring idiopathic pulmonary fibrosis. *European radiology* 2022(cl3, 9114774).
38. Sverzellati N, Silva M, Seletti V, Galeone C, Palmucci S, Piciucchi S, Vancheri C, Poletti V, Tomassetti S, Karwoski R, Bartholmai BJ. Stratification of long-term outcome in stable idiopathic pulmonary fibrosis by combining longitudinal computed tomography and forced vital capacity. *European Radiology* 2020: 30(5): 2669-2679.
39. Tanaka Y, Suzuki Y, Hasegawa H, Yokomura K, Fukada A, Inoue Y, Hozumi H, Karayama M, Furuhashi K, Enomoto N, Fujisawa T, Nakamura Y, Inui N, Suda T. Standardised 3D-CT lung volumes for patients with idiopathic pulmonary fibrosis. *Respir Res* 2022: 23(1): 142.
40. Thillai M, Oldham JM, Ruggiero A, Kanavati F, McLellan T, Saini G, Johnson SR, Ble FX, Azim A, Ostridge K, Platt A, Belvisi M, Maher TM, Molyneaux PL. Deep Learning-based Segmentation of Computed Tomography Scans Predicts Disease Progression and Mortality in Idiopathic Pulmonary Fibrosis. *American Journal of Respiratory and Critical Care Medicine* 2024: 210(4): 465-472.
41. van den Blink B, Dillingh MR, Ginns LC, Morrison LD, Moerland M, Wijsenbeek M, Trehu EG, Bartholmai BJ, Burggraaf J. Recombinant human pentraxin-2 therapy in patients with idiopathic pulmonary fibrosis: safety, pharmacokinetics and exploratory efficacy. *Eur Respir J* 2016: 47(3): 889-897.
42. Walsh SLF, Mackintosh JA, Calandriello L, Silva M, Sverzellati N, Larici AR, Humphries SM, Lynch DA, Jo HE, Glaspole I, Grainge C, Goh N, Hopkins PMA, Moodley Y, Reynolds PN, Zappala C, Keir G, Cooper WA, Mahar AM, Ellis S, Wells AU, Corte TJ. Deep Learning-based Outcome Prediction in Progressive Fibrotic Lung Disease Using High-Resolution Computed Tomography. *Am J Respir Crit Care Med* 2022: 206(7): 883-891.
43. Wang Z, Zhang Z, Zhu L, Hou J, Fu H, Yang X, Wang F, Chen J. Identification of risk factors for acute exacerbation of idiopathic pulmonary fibrosis based on baseline high-resolution computed tomography: a prospective observational study. *BMC Pulmonary Medicine* 2024: 24(1): 352.
44. Wong A, Lu J, Dorfman A, McInnis P, Famouri M, Manary D, Lee JRH, Lynch M. Fibrosis-Net: A Tailored Deep Convolutional Neural Network Design for Prediction of Pulmonary Fibrosis Progression From Chest CT Images. *Front Artif Intell* 2021: 4: 764047.
