# Supplementary material for: A systematic review of the role of quantitative CT in the prognostication and disease monitoring of interstitial lung disease
Source: Eur Respir Rev. 2025 Apr 30;34(176):240194. doi: 10.1183/16000617.0194-2024 (PMC12041933; doi:10.1183/16000617.0194-2024)
Supplement: Supplementary file 5 [file ERR-0194-2024.SUPPLEMENT5.pdf]

Supplementary Table S3 Summary data for journal articles where primary interstitial lung disease subtype was connective tissue disease related interstitial lung disease

Author	Year	Study design	Prognostication	Disease monitoring	CTD-ILD Subtype (s)	Total number of participants included in analysis	Quantitative CT modality	Quantitative CT feature	Prognostication			Disease monitoring		
									Reported outcome statistic	Duration of follow up	Multivariate adjustments	Reported outcome statistic	Interval	Correlation
Amorim *et al*[1]	2024	Retrospective study (Proprietary)	✓	X	SSc	71	CALIPER	Reticulation %	HR 2.70, 95%CI 1.26–5.82 for all-cause mortality	24 months	Age, sex, ground glass, >5% reduction FVC			
Aoki *et al*[2]	2024	Retrospective study (Proprietary)	✓	X	Anti-synthetase	68	QZIP-ILD	Consolidation	OR 1.11 (0.902-1.367 p = 0.325) for response to initial treatment	18 months	Disease duration, FVC%			
Bocchino *et a[3]l*	2019	Primary prospective study	✓	✓	SSc	83	Histogram	Computerized integrated index	Logistic regression for 15% DLCO decline OR 0.31 (0.1-0.96) p=0.042	12 months	None	Pearson correlation coefficient ΔCII vs ΔTLC	12 months	r = 0.45, p= 0.004
Bruni *et al*[4]	2022	Retrospective study (Proprietary)	X	✓	SSc	79	CALIPER Lung vascular software	ILD_EXT				Numerical association only between increasing ΔILD_EXT and ΔFVC	24 months	n/a
Castillo Saldana *et al*[5]	2020	Retrospective study (Proprietary)	✓	✓	SSc	170	Histogram	MLA	Multivariable linear regression showed no association of qCT with survival	Median 5.6 years	Baseline age, sex, pack-years, and lung function	ΔMLA vs ΔDLCO	Median 5.6 years	r =-0.11 (-0.15 – -0.06, p<0.001)
Chassagnon *et al*[6]	2021	Retrospective study (Proprietary)	X	✓	SSc	212	Elastic registration	Mean log_jac				Spearman correlation coefficient mean Δlog_jac vs ΔDLCO%	37 months	R =-0.42 (-0.27--0.54) p<0.001
Clukers *et al*[7]	2021	Retrospective study (Proprietary)	X	✓	SSc	35	FRI	siRADaw				Least square mean change in siRADaw 8.57%, p=0.011 in moderate	18 months	n/a

												to severe disease vs 2.55% p=0.237 in limited disease		
Di Battista *et al[8]*	2024	Primary prospective study	X	✓	SSc	10	CALIPER	Lung texture analysis				Significant correlation between FVC, DLCO and KCO with Normal lung, ground glass and VRS	12 months	n/a
Ferrazza *et al[9]*	2020	Primary prospective study	✓	X	SSc	66	CALIPER	Ground glass pattern %	Ground glass score >4.5 had RR for 10% DLCO reduction 6.8 (1.6-29.2) p<0.01	12 months	None			
Goldin *et al[10]*	2018	Secondary analysis of prospective study	X	✓	SSc	97	QLF	QILD				ΔQILD vs ΔFVC% predicted	24 months	R=-0.37, p<0.001
Humphries *et al[11]*	2024	Retrospective study (Proprietary)	✓	✓	RA-ILD	339	DTA	DTA fibrosis score	DTA fibrosis score mortality risk HR 1.0 (1.03-1.04, p<0.001) and 1.06 (1.01-1.11, p=0.026)	3.9 years and 9.5 years	Age, sex, BMI, smoking, medications, PFTs, visual CT pattern	DTA fibrosis score vs FVC and DLCO	3.9 years and 9.5 years	r=-0.46 and-0.43, p<0.001
Jacob *et al[12]*	2016	Retrospective study (Proprietary)	✓	X	Range	203	CALIPER	PVV	Multivariate mortality analysis PVV HR = 1.57, (1.35-1.82) p<0.0001	46 months	Age, smoking history, PFTs, visual analysis			
Jacob *et al[13]*	2019	Retrospective study (Proprietary)	✓	X	RA	441	CALIPER	VRS	VRS independently predicted mortality. C statistic = 0.77	Maximum 6 years	Age, Sex, Smoking status			
Jamal *et al[14]*	2024	Retrospective study (Proprietary)	✓	X	Anti-synthetase	49	Syngo	Total lung volume	1% Δ TLV AUC 0.81 for prediction progression	283 days	None			
Khanna *et al[15]*	2015	Secondary analysis of prospective study	✓	X	SSc	93	QLF	QILD	Baseline QILD <20% had lower absolute	12 months	None			

									decline in DLCO% vs >20% at baseline 4.8 vs -4.2, p=0.01					
Kim *et al[16]*	2011	Secondary analysis of prospective study	X	✓	SSc	83	QLF	QLF				Pearson correlation QLF vs FVC r=-0.33, p=0.003	12 months	None
Lee *et al[17]*	2021	Retrospective and prospective	X	✓	RA	60	QLF	QILD				Single point Increase in QILD score correlated with radiologist assessed progression p=0.043	24 months	n/a
Le Gall *et a[18]l*	2023	Retrospective study (Proprietary)	✓	X	SSc	318	Deep-learning algorithm	Extent of ILD	1% increase in baseline ILD% for risk of death at 10 years HR 1.04 (1.01-1.07) p=0.004	94 months	Age, sex, antibody status, NYHA score, SSc subtype			
Martyanov *et al[19]*	2017	Primary prospective study	X	✓	SSc	31	QLF	QILD				Change in QILD in most severe lobe vs DLCO% predicted r=-0.69, p=0.0001	6 months	n/a
Occhipinti *et al[20]*	2019	Retrospective study (Proprietary)	✓	X	SSc	35	CALIPER	TLV	Change in TLV predicted change in composite respiratory endpoint AUC 0.74 (0.54-0.93) p=0.03	26 months	None			
Ohno *et al[21]*	2022	Retrospective training and validation cohort Prospective test cohort	X	✓	Range	194	Lung Parenchyma Analysis	Texture analysis				Stepwise regression analysis DVC% vs Dnormal lung/reticulation/honeycombing	>2 years	r2=0.42 p=0.01

Qin *et al[22]*	2023	Retrospective study (Proprietary)	✓	X	Range	215	RAD-Score	3 radiomic features combined in RAD-Score	Combined clinical and radiomic model for prediction overall survival C-index = 0.8, 0.738 and 0.742 in 3 cohorts	34.9 months and 41.7 months	n/a			
Salaffi *et al[23]*	2020	Retrospective study (Proprietary)	✓	X	SSc	45	Histogram	CaM	CaM more responsive than CoVR for detecting disease progression. AUC difference 0.144 (0.0402-0.248) p=0.0065	12 months	n/a			
Schniering *et al[24]*	2022	Secondary analysis of prospective study	✓	X	SSc	156	qRISSC score	26 radiomic features	qRISSc for prediction of progression free survival HR 4.23 (2.03-8.83) p<0.001 Model c-index = 0.75	Not given	Age, Sex, baseline FVC, diffused cutaneous skin involveme nt			
Temiz Karadag *et al[25]*	2021	Retrospective study (Proprietary)	✓	X	SSc	135	Histogram, Myrian XP	Lung Density	Baseline lung density higher in group developed pulmonary fibrosis at follow up. Right lung Density p=0.018 Left lung density p=0.014	10 years +/- 5 years	None			
Ungprasert *et al[26]*	2017	Retrospective study (Proprietary)	X	✓	Idiopathic inflamma tory myopathi es	110	CALIPER	Total interstitial abnormality				ΔTotal interstitial abnormality % vs ΔDLCO	12 months	r=-0.293, p=0.05
Venerito *et al[27]*	2022	Retrospective study (Proprietary)	✓	X	RA	30	Histogram	Median grey level intensity	Prediction of survival HR 9.35 (1.56-55.86)	37.99 months	None			
Volkmann *et al[28]*	2014	Secondary analysis of prospective study	X	✓	SSc	82	QLF	QLF-ZM				Composite of FVC%, QLF-ZM,	12 months	n/a

												HAQ-DI and TDI at 12 months had significant treatment effect favouring cyclophosphamide p=0.001		
Wada *et al[29]*	2020	Retrospective study (Proprietary)	X	✓	SSc	33	YACTA Textural Analysis	Lung density				Post AHSCT FVC correlated with 90th percentile lung density	18 months	r=-0.6676, p<0.05
Wang *et al[30]*	2021	Retrospective study (Proprietary)	✓	X	Anti-MDA5	101	ELVAR	ELVAR	HR for survival 0.098, 0.017-0.564, p=0.009	12 months	Multiple factors (see reference)			
Xu *et al[31]*	2021	Retrospective study (Proprietary)	✓	X	Anti-MDA5	228	CT Pneumonia Analysis	% Consolidation	Prediction of 6 month mortality AI score C-index = 0.78, 0.72-0.85 in derivation, 0.77, 0.64-0.90 for validation	6 months	n/a			
Xu *et al[32]*	2021	Retrospective study (Proprietary)	✓	X	Anti-MDA5	173	RAD-score	RAD-score plus model	Prediction of 6 month mortality C index 0.84, 0.64-1.0 in external validation cohort	6 months	Multiple factors (see reference)			
Yamaguchi *et al[33]*	2022	Retrospective study (Proprietary)	✓	X	Anti-MDA5	34	CT Pneumonia Analysis	Lung severity score	Lung severity score at follow up had AUC 0.844, p<0.01 for prediction of death	Median 35 days	n/a			

AHSCT = Autologous hematopoietic stem cell transplantation, AUC = Area under the curve, CALIPER = Computer-Aided Lung Informatics for Pathology Evaluation and Rating, CaM = Computer aided method, CoVR = Conventional visual reader based score, DLCO = Diffusion capacity of lung for carbon monoxide, ELVAR = Effective lung ventilation area ratio, ILD_EXT = Interstitial lung disease extent, HAQ-DI = Health Assessment Questionnaire-Disability Index, NYHA = New York heart association, MLA = Mean lung attenuation, PFTs = Pulmonary function tests, PVV = pulmonary vessel volume, QLF = Quantitative Lung Fibrosis, QLF-ZM = quantitative lung fibrosis in the zone of maximum fibrosis, QILD = quantitative interstitial lung disease, RA = Rheumatoid arthritis, RR = relative risk, siRADaw = specific image-based airway radius , SSc = Systemic sclerosis, TDI = Transitional dyspnea index, VRS = Vessel related structures, TLC = Total Lung Capacity, TLV = Total lung volume

1. Amorim FG, dos Santos ER, Verrastro CGY, Kayser C. Quantitative chest computed tomography predicts mortality in systemic sclerosis: A longitudinal study. *PLoS ONE* 2024: 19(9 September): e0310892.
2. Aoki R, Iwasawa T, Utsunomiya D, Yamakawa H, Kitamura H, Baba T, Ogura T. Interstitial lung disease associated with anti-aminoacyl-tRNA synthetase syndrome: quantitative evaluation of CT after initial treatment and long-term follow-up. *Acta Radiologica* 2024((Aoki, Iwasawa, Utsunomiya) Department of Diagnostic Radiology, Yokohama City University Graduate School of Medicine, Yokohama-shi, Kanagawa, Japan(Aoki) Department of Diagnostic Radiology, Yokohama City University Medical Center, Kanagawa, Yokohama-shi).
3. Bocchino M, Bruzzese D, D'Alto M, Argiento P, Borgia A, Capaccio A, Romeo E, Russo B, Sanduzzi A, Valente T, Sverzellati N, Rea G, Vettori S. Performance of a new quantitative computed tomography index for interstitial lung disease assessment in systemic sclerosis. *Sci Rep* 2019: 9(1): 9468.
4. Bruni C, Occhipinti M, Pienn M, Camiciottoli G, Bartolucci M, Bosello SL, Payer C, Balint Z, Larici AR, Tottoli A, Tofani L, De Lorenzis E, Lepri G, Bellando-Randone S, Spinella A, Giuggioli D, Masini F, Cuomo G, Lavorini F, Colagrande S, Olschewski H, Matucci-Cerinic M. Lung vascular changes as biomarkers of severity in systemic sclerosis-associated interstitial lung disease. *Rheumatology (Oxford, England)* 2022(100883501, ddb).
5. Castillo Saldana D, Hague CJ, Murphy D, Coxson HO, Tschirren J, Peterson S, Sieren JP, Kirby M, Ryerson CJ, Saldana DC. Association of Computed Tomography Densitometry with Disease Severity, Functional Decline, and Survival in Systemic Sclerosis-associated Interstitial Lung Disease. *Annals of the American Thoracic Society* 2020: 17(7): 813-820.
6. Chassagnon G, Vakalopoulou M, Regent A, Sahasrabudhe M, Marini R, Hoang-Thi TN, Dinh-Xuan AT, Dunogue B, Mouthon L, Paragios N, Revel MP. Elastic Registration-driven Deep Learning for Longitudinal Assessment of Systemic Sclerosis Interstitial Lung Disease at CT. *Radiology* 2021: 298(1): 189-198.
7. Clukers J, Lanclus M, Belmans D, Van Holsbeke C, De Backer W, Vummidi D, Cronin P, Lavon BR, De Backer J, Khanna D. Interstitial lung disease in systemic sclerosis quantification of disease classification and progression with high-resolution computed tomography: An observational study. *J Scleroderma Relat Disord* 2021: 6(2): 154-164.
8. Di Battista M, Delle Sedie A, Romei C, Tavanti L, Da Rio M, Morganti R, Della Rossa A, Mosca M. Lung ultrasound and high-resolution computed tomography quantitative variations during nintedanib treatment for systemic sclerosis-associated interstitial lung disease. *Rheumatology* 2024: 63(11): 3091-3097.
9. Ferrazza AM, Gigante A, Gasperini ML, Ammendola RM, Paone G, Carbone I, Rosato E. Assessment of interstitial lung disease in systemic sclerosis using the quantitative CT algorithm CALIPER. *Clin Rheumatol* 2020: 39(5): 1537-1542.
10. Goldin JG, Kim GHJ, Tseng CH, Volkmann E, Furst D, Clements P, Brown M, Roth M, Khanna D, Tashkin DP. Longitudinal Changes in Quantitative Interstitial Lung Disease on Computed Tomography after Immunosuppression in the Scleroderma Lung Study II. *Ann Am Thorac Soc* 2018: 15(11): 1286-1295.
11. Humphries SM, Adegunsoye A, Demoruelle MK, Wei Kam ML, Amigues I, Bang TJ, Teague SD, Lynch DA, Chung JH, Strek ME, Swigris JJ, Solomon JJ. Quantitative Computed Tomography Analysis in Rheumatoid Arthritis-Related Interstitial Lung Disease. *Chest* 2024(0231335, d1c).
12. Jacob J, Bartholmai BJ, Rajagopalan S, Brun AL, Egashira R, Karwoski R, Kokosi M, Wells AU, Hansell DM. Evaluation of computer-based computer tomography stratification against outcome models in connective tissue disease-related interstitial lung disease: a patient outcome study. *BMC Med* 2016: 14(1): 190.
13. Jacob J, Hirani N, van Moorsel CHM, Rajagopalan S, Murchison JT, van Es HW, Bartholmai Brian J, van Beek FT, Struik MHL, Stewart GA, Kokosi M, Egashira R, Brun AL, Cross G, Barnett J, Devaraj A, Margaritopoulos G, Karwoski R, Renzoni E, Maher TM, Wells Athol U. Predicting outcomes in rheumatoid arthritis related interstitial lung disease. *European Respiratory Journal* 2019: 53(1): 1800869.
14. Jamal F, Shashi K, Vaz N, Doyle T, Dellaripa P, Hammer M. Quantitative Chest Computed Tomography for Progression of Interstitial Lung Disease in Antisynthetase Patients. *Journal of Thoracic Imaging* 2024: 39(5): 281-284.
15. Khanna D, Nagaraja V, Tseng CH, Abtin F, Suh R, Kim G, Wells A, Furst DE, Clements PJ, Roth MD, Tashkin DP, Goldin J. Predictors of lung function decline in scleroderma-related interstitial lung disease based on high-resolution computed tomography: implications for cohort enrichment in systemic sclerosis-associated interstitial lung disease trials. *Arthritis Res Ther* 2015: 17: 372.
16. Kim HJ, Brown MS, Elashoff R, Li G, Gjertson DW, Lynch DA, Strollo DC, Kleerup E, Chong D, Shah SK, Ahmad S, Abtin F, Tashkin DP, Goldin JG. Quantitative texture-based assessment of one-year changes in fibrotic reticular patterns on HRCT in scleroderma lung disease treated with oral cyclophosphamide. *Eur Radiol* 2011: 21(12): 2455-2465.
17. Lee JS, Kim GJ, Ha YJ, Kang EH, Lee YJ, Goldin JG, Lee EY. The Extent and Diverse Trajectories of Longitudinal Changes in Rheumatoid Arthritis Interstitial Lung Diseases Using Quantitative HRCT Scores. *J Clin Med* 2021: 10(17).
18. Le Gall A, Hoang-Thi TN, Porcher R, Dunogue B, Berezne A, Guillevin L, Le Guern V, Cohen P, Chaigne B, London J, Groh M, Paule R, Chassagnon G, Vakalopoulou M, Dinh-Xuan AT, Revel MP, Mouthon L, Regent A. Prognostic value of automated assessment of interstitial lung disease on CT in systemic sclerosis. *Rheumatology (Oxford, England)* 2023((Le Gall, Dunogue, Berezne, Guillevin, Le Guern, Cohen, Chaigne, London, Groh, Paule, Mouthon, Regent) Service de medecine interne, Centre de reference maladies auto-immunes et systemiques rares d'ile de France, Hopital Cochin, Paris F-75014, France(Hoang).
19. Martyanov V, Kim GJ, Hayes W, Du S, Ganguly BJ, Sy O, Lee SK, Bogatkevich GS, Schieven GL, Schiopu E, Marangoni RG, Goldin J, Whitfield ML, Varga J. Novel lung imaging biomarkers and skin gene expression subsetting in dasatinib treatment of systemic sclerosis-associated interstitial lung disease. *PLoS One* 2017: 12(11): e0187580.
20. Occhipinti M, Bosello S, Sisti LG, Cicchetti G, de Waure C, Pirronti T, Ferraccioli G, Gremese E, Larici AR. Quantitative and semi-quantitative computed tomography analysis of interstitial lung disease associated with systemic sclerosis: A longitudinal evaluation of pulmonary parenchyma and vessels. *PLoS One* 2019: 14(3): e0213444.
21. Ohno Y, Aoyagi K, Takenaka D, Yoshikawa T, Fujisawa Y, Sugihara N, Hamabuchi N, Hanamatsu S, Obama Y, Ueda T, Hattori H, Murayama K, Toyama H. Machine learning for lung texture analysis on thin-section CT: Capability for assessments of disease severity and therapeutic effect for connective tissue disease patients in comparison with expert panel evaluations. *Acta Radiol* 2022: 63(10): 1363-1373.
22. Qin S, Kang B, Liu H, Ji C, Li H, Zhang J, Wang X. A computed tomography-based radiomics nomogram for predicting overall survival in patients with connective tissue disease-associated interstitial lung disease. *Eur J Radiol* 2023: 165: 110963.
23. Salaffi F, Carotti M, Tardella M, Di Carlo M, Fraticelli P, Fischetti C, Giovagnoni A, Gabrielli A. Computed tomography assessment of evolution of interstitial lung disease in systemic sclerosis: Comparison of two scoring systems. *Eur J Intern Med* 2020: 76: 71-75.
24. Schniering J, Maciukiewicz M, Gabrys HS, Brunner M, Bluthgen C, Meier C, Braga-Lagache S, Uldry AC, Heller M, Guckenberger M, Fretheim H, Nakas CT, Hoffmann-Vold AM, Distler O, Frauenfelder T, Tanadini-Lang S, Maurer B. Computed tomography-based radiomics decodes prognostic and molecular differences in interstitial lung disease related to systemic sclerosis. *Eur Respir J* 2022: 59(5).
25. Temiz Karadag D, Cakir O, Komac A, Yazici A, Cefle A. Software-based quantitative analysis of lung parenchyma in patients with systemic sclerosis may provide new generation data for pulmonary fibrosis. *Int J Clin Pract* 2021: 75(4): e13931.
26. Ungprasert P, Wilton KM, Ernste FC, Kalra S, Crowson CS, Rajagopalan S, Bartholmai BJ. Novel Assessment of Interstitial Lung Disease Using the "Computer-Aided Lung Informatics for Pathology Evaluation and Rating" (CALIPER) Software System in Idiopathic Inflammatory Myopathies. *Lung* 2017: 195(5): 545-552.

27. Venerito V, Manfredi A, Lopalco G, Lavista M, Cassone G, Scardapane A, Sebastiani M, Iannone F. Radiomics to predict the mortality of patients with rheumatoid arthritis-associated interstitial lung disease: A proof-of-concept study. *Front Med (Lausanne)* 2022: 9: 1069486.
28. Volkmann E, Li N, Tashkin D, Furst D, Elashoff R. Development of a composite outcome measure for systemic sclerosis-related interstitial lung disease. *Annals of the Rheumatic Diseases* 2014: 73(SUPPL. 2).
29. Wada DT, de Almeida FA, de Moraes DA, Dias JBE, Baddini-Martinez J, Oliveira MC, Koenigkam-Santos M. Automatic Quantitative Computed Tomography Evaluation of the Lungs in Patients With Systemic Sclerosis Treated With Autologous Stem Cell Transplantation. *J Clin Rheumatol* 2020: 26(7S Suppl 2): S158-S164.
30. Wang C, Du J, Mei X, Guo L, Li F, Luo H, Li F. The Value of Effective Lung Ventilation Area Ratio Based on CT Image Analysis Is a New Index to Predict the Shorter Outcome of Anti-melanoma Differentiation-Associated Protein 5 Positive Dermatomyositis Associated Interstitial Lung Disease: A Single-Center Retrospective Study. *Front Med (Lausanne)* 2021: 8: 728487.
31. Xu W, Wu W, Zheng Y, Chen Z, Tao X, Zhang D, Zhao J, Wang K, Guo B, Luo Q, Han Q, Zhou Y, Ye S. A Computed Tomography Radiomics-Based Prediction Model on Interstitial Lung Disease in Anti-MDA5-Positive Dermatomyositis. *Front Med (Lausanne)* 2021: 8: 768052.
32. Xu W, Wu W, Zhang D, Chen Z, Tao X, Zhao J, Wang K, Wang X, Zheng Y, Ye S. A novel CT scoring method predicts the prognosis of interstitial lung disease associated with anti-MDA5 positive dermatomyositis. *Sci Rep* 2021: 11(1): 17070.
33. Yamaguchi K, Nakajima T, Yamaguchi A, Itai M, Onuki Y, Shin Y, Uno S, Muto S, Kouno S, Yatomi M, Aoki-Saito H, Hara K, Endo Y, Motegi SI, Muro Y, Nakasatomi M, Sakairi T, Hiromura K, Katsumata N, Hirasawa H, Tsushima Y, Maeno T. Quantitative CT analysis of interstitial pneumonia in anti-melanoma differentiation-associated gene 5 antibody-positive dermatomyositis: a single center, retrospective study. *Clin Rheumatol* 2022: 41(5): 1473-1481.
