# Supplementary material for: A systematic review of the role of quantitative CT in the prognostication and disease monitoring of interstitial lung disease
Source: Eur Respir Rev. 2025 Apr 30;34(176):240194. doi: 10.1183/16000617.0194-2024 (PMC12041933; doi:10.1183/16000617.0194-2024)
Supplement: Supplementary file 6 [file ERR-0194-2024.SUPPLEMENT6.pdf]

Supplementary Table S4 Summary data for journal articles for other interstitial lung disease subtypes

Author	Year	Study design	Prognostication	Disease monitoring	ILD-Subtype	Total number of participants included in analysis	Quantitative CT modality	Quantitative CT feature	Prognostication			Disease monitoring		
									Reported outcome statistic	Duration of follow up	Multivariate adjustments	Reported outcome statistic	Interval	Correlation
Ando *et al[1]*	2013	Retrospective study (Proprietary)	X	✓	CPFE	65	Histogram	Parenchymal density				ΔParenchymal density correlated vs ΔFVC change	1-3 years	r=-0.714, p=0.047
Nemoto *et al[2]*	2020	Retrospective study (Proprietary)	✓	X	CPFE	228	CALIPER	%Fibrosis	Composite endpoint death or progression HR 6.85 (2.85-15.89) p<0.001	Not given	Age, sex, smoking pack-years, CCI, lung cancer, connective tissue disease, and IPF			
Suzuki *et al[3]*	2020	Primary prospective study	✓	X	CPFE	46	Histogram	%AA (%LAA + %HAA)	OR 1.2 (1.02-1.54) p=0.029	1087.9 ± 574.5 days.	Age, BMI, Smoking, FVC%, DLCO%			
Zhao *et al[4]*	2023	Retrospective study (Proprietary)	✓	X	CPFE	1010	SuStaIn CPFE Analysis	Matched-CPFE	Kaplein Meier showed reduced survival in matched-CPFE sub-group vs non-IPF CPFE and Fibrosis-dominant CPFE	Not given	n/a			
Aliboni *et al[5]*	2022	Retrospective study (Proprietary)	X	✓	HP	25	CNN	Fibrosis extent				ΔFibrosis extent vs ΔFVC	1.4 years	r2=0.54,p<0.001
Jacob *et al[6]*	2017	Retrospective study (Proprietary)	✓	X	HP	116	CALIPER	PVV	Multivariate cox analyses PVV for prediction mortality HR 1.74 (1.31-2.31) p<0.0001	58 months	Age, gender and CPI			
Jacob *et al[7]*	2017	Retrospective study (Proprietary)	✓	X	HP	98	CALIPER	Reticulation	Multivariate prediction of survival HR 0.9 (0.93-0.97) P<0.0001	69 months	Age, gender, baseline PFTs			
Yazawa *et al[8]*	2024	Retrospective study (Proprietary)	✓	X	HP	60	3D-CT	Total lung volume	Total lung volume <104 + antigen	34 months	None			

									exposure stratifies fHP into groups with different Kaplein-Meier survival times					
Argula *et al[9]*	201 6	Secondary analysis of prospective study	X	✓	LAM	31	Watershed Cyst Quantification	Volume lung occupied by cysts				Δcyst volume/cyst number or cyst size vs ΔFVC,FEV1,VEGF -D	12 month s	No significant correlation
Ko *et al[10]*	202 0	Secondary analysis of prospective study	X	✓	LAM	20	Histogram	Skewness				ΔFEV1 and ΔSkewness	24 month s	R=0.465, p=0.045
Park *et al[11]*	202 3	Retrospectiv e study (Proprietary)	✓	X	ILA	305	AVIEW	Fibrosis extent	Multivariable LASSO for Risk of ILA progression HR 1.12 (1.02-1.23)	11.3 years	Age, sex, smoking status, BMI, FHx, GERD, Visual CT features			
Shiraishi *et al[12]*	202 4	Retrospectiv e study (Proprietary)	X	✓	ILA	277	AIQCT	ILDvol				ILDvol increased in those with new visual identification of ILA vs no ILA adjusted difference 197.2 (145-249.4) p<0.001	5 years	Age, sex, SMI and smoking
Fukada *et al[13]*	202 2	Retrospectiv e study (Proprietary)	✓	X	PPFE	132	3DCT-Volume	Standardise d upper-lobe volume	0.939 (0.907-0.973) p<0.001	40.3 months and 40.4 months	Age, sex, FVC			
Jacob *et al[14]*	201 7	Retrospectiv e study (Proprietary)	✓	X	Unclassifiabl e	95	CALIPER	PVV	Univariate analysis PVV predictor or mortality HR 1.21 (1.09-1.35) p=0.0003	1.27 years	n/a			
Steele *et al[15]*	202 3	Retrospectiv e study (Proprietary)	✓	X	FIP	296	DTA	Quantitative Fibrosis Score	Quantitative fibrosis score prediction of survival HR 1.06 (1.01-1.10) p=0.013	3.9 years	Age, smoking			
Salhofer *et al[16]*	202 4	Retrospectiv e study (Proprietary)	✓	X	NSIP	71	nnU-NET-based framework	Pulmonary fat index	PFI association with mortality HR 2.37	5 years	Age, gender, BMI, smoking, GAP score, FVC, DLCO,			

									(1.03-5.48) p=0.043		cardiovascular comorbidities, T2DM			
Chae *et al[17]*	2023	Retrospective study (Proprietary)	X	✓	Screening or general population	3118	AVIEW Lung Texture ILA	ILA quantitative assessment				Δquantitative ILA change was higher in group with progressive ILA vs non-progressive ILA	662 days	3.1% vs 0.1%, p<0.001
Choi *et al* [18]	2020	Secondary analysis of prospective study	✓	X	Screening or general population	6671	Histogram	Regional HAA%	Doubling basilar HAA% for prediction of survival = HR 3.98 (2.1-7.55) p<0.001	~ 10 years	Age, sex, ethnicity, BMI, waist circumference, smoking, educational attainment, renal function, total volume of imaged lung, % emphysema, mA dose and study site			
Oh *et al[19]*	2024	Retrospective (proprietary)	✓	X	Screening or general population	2890	QLF	QLF to define ILA	QLF defined ILA progression vs stability had poorer survival time 115.7 months vs 118.1 months p=0.013	5 years	Not applicable			
Aoki *et al[20]*	2022	Primary prospective study	✓	X	Combined subtypes	214	GHNC	CF_{DL}	CF_{DL} for the prediction of survival = HR=1.477 (1.277–1.708) p<0.001	3.3 years	Age, sex			
Alkhanfar *et al[21]*	2022	Retrospective study (Proprietary)	✓	X	Combined subtypes	122	Apollo v2.0 pulmonary vessel segmentation	Pulmonary vessels	Pulmonary vessels <0.8mm for prediction survival HR 0.92, p=0.03	Not given	Age, sex			
Arzhaeva *et al[22]*	2010	Retrospective study (Proprietary)	X	✓	Combined subtypes	75	Histogram	Dissimilarity-based features and intensity distribution features				qCT had a accuracy of 79.5% for classification into regression, progression or unchanged	1 month to 2 years	n/a

Barros *et al[23]*	2022	Retrospective study (Proprietary)	✓	X	Combined subtypes	306	Histogram	NLI	NLI <70% for prediction of mortlity HR 2.72, p=0.005	33 months	Age, sex			
Crews *et al [24]*	2020	Retrospective study (Proprietary)	✓	X	Combined subtypes	225	CALIPER	VRS	VRS multivariate analysis for mortality coefficient 0.008 (0-0.015) p=0.041	Not given	Age, sex			
Gudmundsson *et al[25]*	2023	Retrospective study (Proprietary)	✓	✓	Combined subtypes	414	cPPFE	%Visceral pleural surface affected by PPFE	Δ-PPFE independently associated with mortality HR 1.2 (1.16-1.34) p<0.0001	2.2 and 2.7 years	Age, sex, smoking, emphysema, antifibrotic use and DLCO	ΔPPFE vs ΔFVC	2.2 and 2.7 years	R^2 = 0.07, p<0.0001
Guerra *et al[26]*	2023	Retrospective study (Proprietary)	✓	✓	Combined subtypes	188	U-Net CNN	%ILD	4% Progression rate had AUC 0.83, p<0.001 for prediction of mortality	3.8 years	n/a	ΔILD vs ΔFVC	3.8 years	ρ = -0.30, -0.16 –0.45, p=0.004
Humphries *et al[27]*	2024	Retrospective study (Online and Proprietary)	✓	X	Combined subtypes	1218	MIL-UIP	MIL-UIP score	MIL-UIP classification prediction of mortality HR 5.09 (1.94-13.4) p=<0.001 and 2.65 (1.7-4.8) p=0.001	6 years	Age, sex, smoking history			
Iwasawa *et al[28]*	2017	Retrospective study (Proprietary)	✓	X	Combined subtypes	79	Histogram	Subpleural fibrotic lesion volume “H-pattern”	H pattern for prediction of mortality HR 4.144, (1.385-12.397) p=0.011	59.7 months	Age			
Karampitsakos *et al* [29]	2022	Retrospective study (Proprietary)	X	✓	Combined subtypes	55	CALIPER	GGO, HCM and reticulation				Interclass correlation between CALIPER and visual ΔGGO, Δreticulation and ΔHCM	1 year	0.73, 0.88 and 0.6
Koh *et al[30]*	2024	Retrospective (Proprietary)	✓	✓	Combined subtypes	468	AVIEW Lung texture	Fibrosis and total ILD extent	Total ILD extent HR 2.484 (1.565-3.943) for prediction of	75 months	Baseline FVC	Total ILD extent vs Absolute decline FVC	44 months	r=0.24

									5% absolute decline FVC					
Matsuoka *et al[31]*	201 5	Retrospectiv e study (Proprietary)	X	✓	Combined subtypes	112	Histogram	DA% (%LAA + %HAA)				ΔDA% in CPFE vs emphysema vs ILD	5 years	12.9% vs 4.9% vs 7.1%, p<0.0001
Mei *et al[32]*	202 3	Retrospectiv e study (Proprietary)	✓	X	Combined subtypes	449	CT-based CNN model	Transformer model	1 year transformer model to predict 3 year survival AOC = 0.632 (0.413-0.851) p=0.05	3 years	None			
Moon *et al[33]*	201 6	Primary prospective study	✓	X	Combined subtypes	28	CADLAB Features and perfusion based CT measurement s	Mean iodine value of total lung volume	Prediction of survival HR 1.19 (1.01-1.40) p=0.04	Not given	Age, sex, diagnosis, GGO%, ILD%			
Moran-Mendoza *et al[34]*	202 4	Retrospectiv e (Online)	✓	X	Combined subtypes	228	Fibresolve	Fibresolve score	Mortality association HR 7.14 (1.31-38.85) p=0.02	2.8 years	Gender, age, FVC predicted, UIP pattern, pulmonary hypertension, smoking, lung cancer, coronary artery disease			
Nan *et al[35]*	202 4	Retrospectiv e (Online)	✓	X	Combined subtypes	312	Multiple	Total airway volume (TAV)	Mortality association HR 1.83 (1.35-2.49) p<0.0001	63 weeks	SOFIA, AvgFib, FVC%			
Oh *et al[36]*	202 3	Retrospectiv e study (Proprietary)	✓	X	Combined subtypes	979	DTA	Fibrosis extent	Fibrosis extent associated with transplant free survival HR 1.04 (1.04-1.05) p<0.001	3 years	Age, sex, BMI and smoking			
Selvan *et al[37]*	202 4	Retrospectiv e (Online)	✓	X	Combined subtypes	643	Fibresolve	Fibresolve score tertile	Association with mortality Fibresolve Tertile 3 HR 3.12 (1.98-4.90) p<0.001	144 weeks	Age, sex, FVC, tobacco use, modified GAP			
Shin *et al[38]*	202 4	Retrospectiv e (Online)	✓	X	Combined subtypes	131	Aview	Reticulation	Reticulation + KL-6 levels AUC 0.810 (0.646-0.973) p=0.001 for	13 months	None			

									prediction of respiratory hospitalisation					
Si-Mohamed *et al[39]*	2022	Retrospective study (Proprietary)	✓	X	Combined subtypes	424	CT Pulmo Auto	Relative annual CTVol loss	Univariate Cox regression model for prediction of survival HR 6.8 (2.3-20) p=0.0006	3 years	None			
Sumikawa *et al[40]*	2006	Retrospective study (Proprietary)	X	✓	Combined subtypes	38	Histogram	CNT, VAR and EPY				CNT, VAR and EPY pre- and post treatment	2.8 months	CNT higher and VAR and EPY lower post treatment $p<0.05$
Tanizawa *et al[41]*	2015	Retrospective study (Proprietary)	✓	X	Combined subtypes	74	Histogram	Kurtosis	Kurtosis predictor of mortality HR 0.67 (0.44-0.96) p=0.03	>3 months	Age, sex, diagnosis (separately)			
Umakoshi *et al[42]*	2019	Retrospective study (Proprietary)	✓	X	Combined subtypes	45	3D-cHRCT	%HAA at 20mm	Univariate logistic regression association with physiological progression %HAA at 20mm OR 13.6, p=0.15	210 days	None			
Yoon *et al[43]*	2013	Retrospective study (Proprietary)	✓	✓	Combined subtypes	89	Automated quantification system	Fibrosis score				ΔFibrosis score correlated with ΔFVC	1 year	r=-0.465, $p<0.05$
Zou *et al[44]*	2023	Secondary analysis of prospective study	✓	✓	Combined subtypes	271	CALIPER	All CALIPER HRCT metrics	Adjusted R^2 for FVC slope response variable <0.2	28-52 weeks	Baseline FVC, age, gender, height			

AA = Abnormal area, BMI = Body mass index, CALIPER = Computer-Aided Lung Informatics for Pathology Evaluation and Rating, CCI = Charlson comorbidity index, CFDL = Consolidation with fibrosis, CNN = Convolutional neural network, CNT = contrast, CPFE = Combined pulmonary fibrosis and emphysema, CPI = Composite physiological index, DA = Destructed lung area, DTA = Data driven textural analysis, EPY = entropy, FEV1 = Forced expiration volume in 1 second, FHx = Family history, FIP = Familial interstitial pneumonia, FVC = Forced vital capacity, GERD = Gastro-esophageal reflux disease, GGO = ground glass opacities, GHNC = Gaussian histogram normalized correlation, HAA = High attenuation Area, HCM = Honeycombing, HR = Hazard ratio, ILA = Interstitial lung abnormality, ILD = Interstitial lung disease, IPF = Idiopathic pulmonary fibrosis, LAA = Low attenuation area, NLI = Normal lung index, NSIP = Non-specific interstitial pneumonitis, PFPE = Pleuroparenchymal fibroelastosis, PVV = pulmonary vessel volume, SVV = small vessel volume, T2DM = Type 2 Diabetes Mellitus, VAR = variance, VEGF-D = Vascular endothelial growth factor-D, VRS = Vessel related structures

1. Ando K, Sekiya M, Tobino K, Takahashi K. Relationship between quantitative CT metrics and pulmonary function in combined pulmonary fibrosis and emphysema. *Lung* 2013: 191(6): 585-591.
2. Nemoto M, Nei Y, Bartholmai B, Yoshida K, Matsui H, Nakashita T, Motojima S, Aoshima M, Ryu JH. Automated computed tomography quantification of fibrosis predicts prognosis in combined pulmonary fibrosis and emphysema in a real-world setting: a single-centre, retrospective study. *Respir Res* 2020: 21(1): 275.
3. Suzuki M, Kawata N, Abe M, Yokota H, Anazawa R, Matsuura Y, Ikari J, Matsuoka S, Tsushima K, Tatsumi K. Objective quantitative multidetector computed tomography assessments in patients with combined pulmonary fibrosis with emphysema: Relationship with pulmonary function and clinical events. *PLoS One* 2020: 15(9): e0239066.

4. Zhao A, Gudmundsson E, Mogulkoc N, van Moorsel C, Corte TJ, Vasudev P, Romei C, Chapman R, Wallis TJM, Denneny E, Goos T, Savas R, Ahmed A, Brereton CJ, van Es HW, Jo H, De Liperi A, Duncan M, Pontoppidan K, De Sadeleer LJ, van Beek F, Barnett J, Cross G, Procter A, Veltkamp M, Hopkins P, Moodley Y, Taliani A, Taylor M, Verleden S, Tavanti L, Vermant M, Nair A, Stewart I, Janes SM, Young AL, Barber D, Alexander DC, Porter JC, Wells AU, Jones MG, Wuyts WA, Jacob J. Mortality surrogates in combined pulmonary fibrosis and emphysema. *Eur Respir J* 2023.
5. Aliboni L, Dias OM, Baldi BG, Sawamura MVY, Chate RC, Carvalho CRR, de Albuquerque ALP, Aliverti A, Pennati F. A Convolutional Neural Network Approach to Quantify Lung Disease Progression in Patients with Fibrotic Hypersensitivity Pneumonitis (HP). *Acad Radiol* 2022: 29(8): e149-e156.
6. Jacob J, Bartholmai BJ, Egashira R, Brun AL, Rajagopalan S, Karwoski R, Kokosi M, Hansell DM, Wells AU. Chronic hypersensitivity pneumonitis: identification of key prognostic determinants using automated CT analysis. *BMC Pulm Med* 2017: 17(1): 81.
7. Jacob J, Bartholmai BJ, Rajagopalan S, Karwoski R, Mak SM, Mok W, Della Casa G, Sugino K, Walsh SLF, Wells AU, Hansell DM. Automated computer-based CT stratification as a predictor of outcome in hypersensitivity pneumonitis. *Eur Radiol* 2017: 27(9): 3635-3646.
8. Yazawa S, Suzuki Y, Tanaka Y, Yokomura K, Kono M, Hashimoto D, Fukada A, Inoue Y, Yasui H, Hozumi H, Karayama M, Furuhashi K, Enomoto N, Fujisawa T, Inui N, Suda T. 3D-CT-derived lung volumes and mortality risk in patients with fibrotic hypersensitivity pneumonitis. *Allergology International* 2024((Yazawa, Suzuki, Tanaka, Fukada, Inoue, Yasui, Hozumi, Karayama, Furuhashi, Enomoto, Fujisawa, Inui, Suda) Second Division, Department of Internal Medicine, Hamamatsu University School of Medicine, Hamamatsu, Japan(Yokomura) Department of Respiratory Medi).
9. Argula RG, Kokosi M, Lo P, Kim HJ, Ravenel JG, Meyer C, Goldin J, Lee HS, Strange C, McCormack FX, Investigators MS. A Novel Quantitative Computed Tomographic Analysis Suggests How Sirolimus Stabilizes Progressive Air Trapping in Lymphangioleiomyomatosis. *Ann Am Thorac Soc* 2016: 13(3): 342-349.
10. Ko Y, Asakawa K, Tobino K, Oguma T, Hirai T, Takada T, Takahashi K, Seyama K, Multicenter Lymphangioleiomyomatosis Sirolimus Trial for Safety Study G. Quantitative analysis of computed tomography of the lungs in patients with lymphangioleiomyomatosis treated with sirolimus. *Heliyon* 2020: 6(2): e03345.
11. Park S, Choe J, Hwang HJ, Noh HN, Jung YJ, Lee JB, Do KH, Chae EJ, Seo JB. Long-Term Follow-Up of Interstitial Lung Abnormality: Implication in Follow-Up Strategy and Risk Thresholds. *Am J Respir Crit Care Med* 2023: 208(8): 858-867.
12. Shiraishi Y, Tanabe N, Sakamoto R, Maetani T, Kaji S, Shima H, Terada S, Terada K, Ikezoe K, Tanizawa K, Oguma T, Handa T, Sato S, Muro S, Hirai T. Longitudinal assessment of interstitial lung abnormalities on CT in patients with COPD using artificial intelligence-based segmentation: a prospective observational study. *BMC pulmonary medicine* 2024: 24(1): 200.
13. Fukada A, Suzuki Y, Mori K, Kono M, Hasegawa H, Hashimoto D, Yokomura K, Imokawa S, Tanaka Y, Inoue Y, Hozumi H, Karayama M, Furuhashi K, Enomoto N, Fujisawa T, Nakamura Y, Inui N, Fujino Y, Nakamura H, Suda T. Idiopathic pleuroparenchymal fibroelastosis: three-dimensional computed tomography assessment of upper-lobe lung volume. *Eur Respir J* 2022: 60(6).
14. Jacob J, Bartholmai BJ, Rajagopalan S, Egashira R, Brun AL, Kokosi M, Nair A, Walsh SLF, Karwoski R, Nicholson AG, Hansell DM, Wells AU. Unclassifiable-interstitial lung disease: Outcome prediction using CT and functional indices. *Respir Med* 2017: 130: 43-51.
15. Steele MP, Peljto AL, Mathai SK, Humphries S, Bang TJ, Oh A, Teague S, Cicchetti G, Sigakis C, Kropski JA, Loyd JE, Blackwell TS, Brown KK, Schwarz MI, Warren RA, Powers J, Walts AD, Markin C, Fingerlin TE, Yang IV, Lynch DA, Lee JS, Schwartz DA. Incidence and Progression of Fibrotic Lung Disease in an At-Risk Cohort. *Am J Respir Crit Care Med* 2023: 207(5): 587-593.
16. Salhofer L, Bonella F, Meetschen M, Umutlu L, Forsting M, Schaarschmidt BM, Opitz M, Beck N, Zensen S, Hosch R, Parmar V, Nensa F, Haubold J. CT-based body composition analysis and pulmonary fat attenuation volume as biomarkers to predict overall survival in patients with non-specific interstitial pneumonia. *European Radiology Experimental* 2024: 8(1): 114.
17. Chae KJ, Lim S, Seo JB, Hwang HJ, Choi H, Lynch D, Jin GY. Interstitial Lung Abnormalities at CT in the Korean National Lung Cancer Screening Program: Prevalence and Deep Learning-based Texture Analysis. *Radiology* 2023: 307(4): e222828.
18. Choi B, Kawut SM, Raghu G, Hoffman E, Tracy R, Madahar P, Bernstein EJ, Barr RG, Lederer DJ, Podolanczuk A. Regional distribution of high-attenuation areas on chest computed tomography in the Multi-Ethnic Study of Atherosclerosis. *ERJ Open Res* 2020: 6(1).
19. Oh JH, Kim GHJ, Song JW. Interstitial lung abnormality evaluated by an automated quantification system: prevalence and progression rate. *Respiratory Research* 2024: 25(1).
20. Aoki R, Iwasawa T, Saka T, Yamashiro T, Utsunomiya D, Misumi T, Baba T, Ogura T. Effects of Automatic Deep-Learning-Based Lung Analysis on Quantification of Interstitial Lung Disease: Correlation with Pulmonary Function Test Results and Prognosis. *Diagnostics (Basel)* 2022: 12(12): 3038.
21. Alkhanfar D, Shahin Y, Alandejani F, Dwivedi K, Alabed S, Johns C, Lawrie A, Thompson AAR, Rothman AMK, Tschirren J, Uthoff JM, Hoffman E, Condliffe R, Wild JM, Kiely DG, Swift AJ. Severe pulmonary hypertension associated with lung disease is characterised by a loss of small pulmonary vessels on quantitative computed tomography. *ERJ Open Res* 2022: 8(2).
22. Arzhaeva Y, Prokop M, Murphy K, van Rikxoort EM, de Jong PA, Gietema HA, Viergever MA, van Ginneken B. Automated estimation of progression of interstitial lung disease in CT images. *Med Phys* 2010: 37(1): 63-73.
23. Barros MC, Hochhegger B, Altmayer S, Zanon M, Sartori G, Watte G, do Nascimento MHS, Chatkin JM. The Normal Lung Index From Quantitative Computed Tomography for the Evaluation of Obstructive and Restrictive Lung Disease. *J Thorac Imaging* 2022: 37(4): 246-252.
24. Crews MS, Bartholmai BJ, Adegunsoye A, Oldham JM, Montner SM, Karwoski RA, Husain AN, Vij R, Noth I, Strek ME, Chung JH. Automated CT Analysis of Major Forms of Interstitial Lung Disease. *J Clin Med* 2020: 9(11).
25. Gudmundsson E, Zhao A, Mogulkoc N, van Beek F, Goos T, Brereton CJ, Veltkamp M, Chapman R, van Es HW, Garthwaite H, Gholipour B, Heightman M, Nair A, Pontoppidan K, Savas R, Ahmed A, Vermant M, Unat O, Procter A, De Sadeleer L, Denneny E, Wallis T, Duncan M, Taylor M, Verleden S, Janes SM, Alexander DC, Wells AU, Porter J, Jones MG, Stewart I, van Moorsel CHM, Wuyts W, Jacob J. Delineating associations of progressive pleuroparenchymal fibroelastosis in patients with pulmonary fibrosis. *ERJ Open Res* 2023: 9(2): 00637-02022.
26. Guerra X, Rennotte S, Fetita C, Boubaya M, Debray MP, Israel-Biet D, Bernaudin JF, Valeyre D, Cadranel J, Naccache JM, Nunes H, Brillet PY. U-net convolutional neural network applied to progressive fibrotic interstitial lung disease: Is progression at CT scan associated with a clinical outcome? *Respir Med Res* 2023: 85: 101058.
27. Humphries SM, Thieke D, Baraghoshi D, Strand MJ, Swigris JJ, Chae KJ, Hwang HJ, Oh AS, Flaherty KR, Adegunsoye A, Jablonski R, Lee CT, Husain AN, Chung JH, Strek ME, Lynch DA. Deep Learning Classification of Usual Interstitial Pneumonia Predicts Outcomes. *American Journal of Respiratory and Critical Care Medicine* 2024: 209(9): 1121-1131.
28. Iwasawa T, Takemura T, Okudera K, Gotoh T, Iwao Y, Kitamura H, Baba T, Ogura T, Oba MS. The importance of subpleural fibrosis in the prognosis of patients with idiopathic interstitial pneumonias. *Eur J Radiol* 2017: 90: 106-113.
29. Karampitsakos T, Kalogeropoulou C, Tzilas V, Papaioannou O, Kazantzi A, Koukaki E, Katsaras M, Bouros E, Tsiri P, Tsirikos G, Zarkadi E, Ntoulias N, Sotiropoulou V, Efthymiou P, Chrysikos S, Malakounidou E, Sampsonas F, Bouros D, Tzouvelekis A. Safety and Effectiveness of Mycophenolate Mofetil in Interstitial Lung Diseases: Insights from a Machine Learning Radiographic Model. *Respiration* 2022: 101(3): 262-271.
30. Koh SY, Lee JH, Park H, Goo JM. Value of CT quantification in progressive fibrosing interstitial lung disease: a deep learning approach. *Eur Radiol* 2024: 34(7): 4195-4205.

31. Matsuoka S, Yamashiro T, Matsushita S, Fujikawa A, Kotoku A, Yagihashi K, Kurihara Y, Nakajima Y. Morphological disease progression of combined pulmonary fibrosis and emphysema: comparison with emphysema alone and pulmonary fibrosis alone. *J Comput Assist Tomogr* 2015: 39(2): 153-159.
32. Mei X, Liu Z, Singh A, Lange M, Boddu P, Gong JQX, Lee J, DeMarco C, Cao C, Platt S, Sivakumar G, Gross B, Huang M, Masseaux J, Dua S, Bernheim A, Chung M, Deyer T, Jacobi A, Padilla M, Fayad ZA, Yang Y. Interstitial lung disease diagnosis and prognosis using an AI system integrating longitudinal data. *Nat Commun* 2023: 14(1): 2272.
33. Moon JW, Bae JP, Lee HY, Kim N, Chung MP, Park HY, Chang Y, Seo JB, Lee KS. Perfusion- and pattern-based quantitative CT indexes using contrast-enhanced dual-energy computed tomography in diffuse interstitial lung disease: relationships with physiologic impairment and prediction of prognosis. *Eur Radiol* 2016: 26(5): 1368-1377.
34. Moran-Mendoza O, Singla A, Kalra A, Muelly M, Reicher JJ. Computed tomography machine learning classifier correlates with mortality in interstitial lung disease. *Respiratory Investigation* 2024: 62(4): 670-676.
35. Nan Y, Xing X, ShiyiWang, Tang Z, Felder FN, Zhang S, Ledda RE, Ding X, Yu R, Liu W, Shi F, Sun T, Cao Z, Zhang M, Gu Y, Zhang H, Gao J, Wang P, Tang W, Yu P, Kang H, Chen J, Lu X, Zhang B, Mamalakis M, Prinzi F, Carlini G, Cuneo L, Banerjee A, Xing Z, Zhu L, Mesbah Z, Jain D, Mayet T, Yuan H, Lyu Q, Qayyum A, Mazher M, Wells A, Walsh SL, Yang G. Hunting imaging biomarkers in pulmonary fibrosis: Benchmarks of the AIIB23 challenge. *Medical Image Analysis* 2024: 97((Nan, Xing, Tang, Yang) Bioengineering Department and Imperial-X, Imperial College London, London, United Kingdom(Nan, Felder, Wells, Walsh, Yang) Royal Brompton Hospital, London, United Kingdom(ShiyiWang, Felder, Zhang, Qayyum, Wells, Walsh, Yang) Nation): 103253.
36. Oh AS, Lynch DA, Swigris JJ, Baraghoshi D, Dyer DS, Hale VA, Koelsch TL, Marrocchio C, Parker KN, Teague S, Flaherty KR, Humphries SM. Deep Learning–based Fibrosis Extent on CT Predicts Outcome of Fibrosing Interstitial Lung Disease Independent of Visually Assessed CT Pattern. *Annals of the American Thoracic Society* 2023.
37. Selvan KC, Reicher J, Muelly M, Kalra A, Adegunsoye A. Machine learning classifier is associated with mortality in interstitial lung disease: a retrospective validation study leveraging registry data. *BMC Pulmonary Medicine* 2024: 24(1): 254.
38. Shin B, Oh YJ, Kim J, Park SG, Lee KS, Lee HY. Correlation between CT-based phenotypes and serum biomarker in interstitial lung diseases. *BMC Pulmonary Medicine* 2024: 24(1): 523.
39. Si-Mohamed SA, Nasser M, Colevray M, Nempont O, Lartaud PJ, Vlachomitrou A, Broussaud T, Ahmad K, Traclet J, Cottin V, Boussel L. Automatic quantitative computed tomography measurement of longitudinal lung volume loss in interstitial lung diseases. *Eur Radiol* 2022: 32(6): 4292-4303.
40. Sumikawa H, Johkoh T, Yamamoto S, Takahei K, Ueguchi T, Ogata Y, Matsumoto M, Fujita Y, Natsag J, Inoue A, Tsubamoto M, Mihara N, Honda O, Tomiyama N, Hamada S, Nakamura H. Quantitative analysis for computed tomography findings of various diffuse lung diseases using volume histogram analysis. *Journal of computer assisted tomography* 2006: 30(2): 244-249.
41. Tanizawa K, Handa T, Nagai S, Hirai T, Kubo T, Oguma T, Ito I, Ito Y, Watanabe K, Aihara K, Ikezoe K, Oga T, Chin K, Izumi T, Mishima M. Clinical impact of high-attenuation and cystic areas on computed tomography in fibrotic idiopathic interstitial pneumonias. *BMC Pulm Med* 2015: 15: 74.
42. Umakoshi H, Iwano S, Inoue T, Li Y, Nakamura K, Naganawa S. Quantitative Follow-Up Assessment of Patients with Interstitial Lung Disease by 3D-Curved High-Resolution CT Imaging Parallel to the Chest Wall. *Nagoya J Med Sci* 2019: 81(1): 41-53.
43. Yoon RG, Seo JB, Kim N, Lee HJ, Lee SM, Lee YK, Song JW, Song JW, Kim DS. Quantitative assessment of change in regional disease patterns on serial HRCT of fibrotic interstitial pneumonia with texture-based automated quantification system. *Eur Radiol* 2013: 23(3): 692-701.
44. Zou Y, Hou X, Anegondi N, Negahdar M, Cheung D, Belloni P, de Crespigny A, Coimbra AF. Weak to no correlation between quantitative high-resolution computed tomography metrics and lung function change in fibrotic diseases. *ERJ Open Res* 2023: 9(5): 00210-02023.
